# Airborne particulate matters induce thrombopoiesis from megakaryocytes through regulating mitochondrial oxidative phosphorylation

**DOI:** 10.1186/s12989-021-00411-4

**Published:** 2021-05-13

**Authors:** Xiaoting Jin, Hongyan Yu, Baoqiang Wang, Zhendong Sun, Ze Zhang, Qian S. Liu, Yuxin Zheng, Qunfang Zhou, Guibin Jiang

**Affiliations:** 1 State Key Laboratory of Environmental Chemistry and Ecotoxicology, Research Center for Eco-Environmental Sciences, Chinese Academy of Sciences, Beijing, 100085 People’s Republic of China; 2 China School of Public Health, Qingdao University, Qingdao, 266071 People’s Republic of China; 3 School of Environment, Hangzhou Institute for Advanced Study, University of Chinese Academy of Sciences, Hangzhou, 310000 People’s Republic of China; 4 College of Resources and Environment, University of Chinese Academy of Sciences, Beijing, 100049 People’s Republic of China; 5 Institute of Environment and Health, Jianghan University, Wuhan, 430056 People’s Republic of China

**Keywords:** Airborne fine particulate matters, Thrombopoiesis, Megakaryocyte, Differentiation, Mitochondrial oxidative phosphorylation

## Abstract

**Background:**

Although airborne fine particulate matter (PM) pollution has been demonstrated as an independent risk factor for pulmonary and cardiovascular diseases, their currently-available toxicological data is still far from sufficient to explain the cause-and-effect. Platelets can regulate a variety of physiological and pathological processes, and the epidemiological study has indicated a positive association between PM exposure and the increased number of circulative platelets. As one of the target organs for PM pollution, the lung has been found to be involved in the storage of platelet progenitor cells (i.e. megakaryocytes) and thrombopoiesis. Whether PM exposure influences thrombopoiesis or not is thus explored in the present study by investigating the differentiation of megakaryocytes upon PM treatment.

**Results:**

The results showed that PM exposure promoted the thrombopoiesis in an exposure concentration-dependent manner. PM exposure induced the megakaryocytic maturation and development by causing cell morphological changes, occurrence of DNA ploidy, and alteration in the expressions of biomarkers for platelet formation. The proteomics assay demonstrated that the main metabolic pathway regulating PM-incurred alteration of megakaryocytic maturation and thrombopoiesis was the mitochondrial oxidative phosphorylation (OXPHOS) process. Furthermore, airborne PM sample promoted-thrombopoiesis from megakaryocytes was related to particle size, but independent of sampling filters.

**Conclusion:**

The findings for the first time unveil the potential perturbation of haze exposure in thrombopoiesis from megakaryocytes by regulating mitochondrial OXPHOS. The substantial evidence on haze particle-incurred hematotoxicity obtained herein provided new insights for assessing the hazardous health risks from PM pollution.

**Supplementary Information:**

The online version contains supplementary material available at 10.1186/s12989-021-00411-4.

## Introduction

In recent years, dust-haze air pollution caused by human activities has frequently plagued vast areas of China, and represented a major burden on public health [1, 2]. The accumulating epidemiological data have revealed the positive relationship between airborne fine particulate matter (PM) exposure and hazardous human health risks, which not only exacerbates the mortality of pulmonary and respiratory diseases, including pneumonia, chronic obstructive pulmonary disease, and lung cancer, [3–5] but also plays a pivotal role in the progression of cardiovascular diseases, such as arrhythmia, myocardial infarction and cardiac hypertrophy [6]. Previous studies mainly focused on the direct toxicological effects on biological target organs, such as heart tissue and lung tissue [7, 8]. However, the currently available data is still far from sufficient to explain the cause-and-effect due to the complex properties of PM [9]. Exploring sensitive biomarkers and the underlying molecular mechanisms are of high importance for understanding PM-induced deleterious health effects.

Platelet has a pivotal role in the maintenance of normal hemostasis and regulation of coagulation, inflammation as well as immune processes, and its disorders are closely related to the incidence of many diseases and their progression, including pulmonary and cardiovascular diseases [10, 11]. It has been reported that activated platelets can interact with both the pulmonary endothelial cells and the white blood cells in the pulmonary blood vessels, thus initiating or worsening the dysfunction and damage of lung tissues [12, 13]. Platelet is also a major factor for the deterioration of cardiovascular diseases, including acute coronary syndrome, stroke, myocardial infarction, and myocardial ischemia [14, 15]. Since the main health risk from airborne PM exposure is pulmonary and cardiovascular diseases, whether the platelet is involved or not is worthy of being studied. The epidemiological investigation on 6488 cases revealed that an increase of 2.4 μg/m^3^ in long-term PM_2.5_ exposure was associated with a relative elevation of 2.3% (95% CI 1.4 to 3.3%) in the number of circulative platelets [16]. Animal studies also showed that both the platelet number and the platelet surface marker of CD41 level were remarkably increased in concentrated PM-exposed mice [17, 18]. Given the critical pathophysiological role of platelets, understanding how airborne fine particles may influence thrombopoiesis would help explain the impacts of haze on human health.

The circulative platelets normally derive from megakaryocytes through a process of maturation, development, and fragmentation of this kind of highly specialized precursor cells [19–21]. There are roughly 1 × 10^11^ platelets produced by the cytoplasmic fragmentation of megakaryocytes each day in human bodies [22]. Besides bone marrow, lung tissue also contributes to megakaryocyte storage and thrombopoiesis [23, 24]. The total number of megakaryocytes in pulmonary tissues is equivalent to that in bone marrow, and the biogenesis of platelets in lung accounts for approximately 50% of total platelet production in whole bodies [23]. As the main deposition organ of the inhaled PMs, [9] lung may thus provide the direct encounter place for these exogenous airborne particles and pulmonary megakaryocytes, thus potentially incurring the abnormal functional changes of these precursor cells like excessive thrombopoiesis.

In the present study, we aimed to investigate the effects of airborne fine particles on thrombopoiesis from megakaryocytes, elucidate the underlying mechanism involved in PM-stimulated megakaryocytic maturation, development, and fragmentation, and illustrate the influences from particle size as well as sampling filter. The findings will provide a novel hematological explanation for haze-induced health impacts.

## Result

### Characterization of QFF-PM_2.5_

**Fig. 1:**
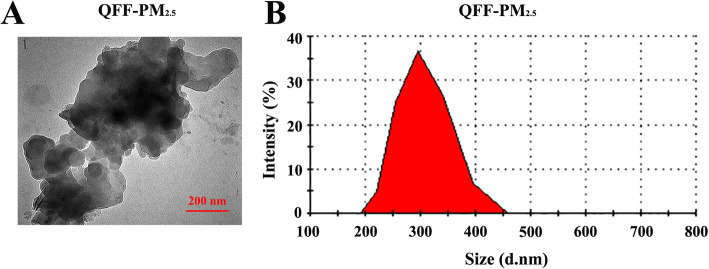
Characterization of QFF-PM_2.5_. **a** Representative TEM images of PM_2.5_ were collected by an inorganic quartz filter (QFF). **b** The hydrodynamic size distribution of QFF-PM_2.5_ in Milli-Q water

The airborne PM sample collected by inorganic quartz filter (QFF-PM_2.5_) used in this study was characterized, and the results showed that its morphology exhibited in irregular particulate aggregates (Fig. 1a), and the hydrodynamic diameters were in the range of 200 nm to 450 nm (Fig. 1b). The information on its source (i.e. sampling location and time) and other physicochemical properties (like zeta potential, chemical components, and endotoxin level) were previously reported, [25] as the same batch of QFF-PM_2.5_ sample was used herein.

### Cell viability and growth of megakaryocytes upon QFF-PM_2.5_ treatment

To evaluate the effect of QFF-PM_2.5_ on thrombopoiesis from megakaryocytes, Dami cell line was used as a sound model for studying the differentiation of human megakaryocytic cells [26]. The non-cytotoxic concentrations of QFF-PM_2.5_ were firstly screened using Alamar Blue assay, and the results based on cell viabilities (Fig. S1A) and morphological observation (Fig. S1B) showed QFF-PM_2.5_ exposure caused no significant cytotoxicity at the concentration of 0.1 μg/mL or lower (*p* > 0.05). Therefore, the concentration of 0.1 μg/mL or lower was considered as the non-cytotoxic level for Dami cells in the current study, and used in the following experiments.

To select the exposure concentration and time for QFF-PM_2.5_ effectively inducing thrombopoiesis from megakaryocytes, the growth curves of megakaryocytes exposed to different concentrations of QFF-PM_2.5_ were monitored on different days. Figure S2 shows the lag phase of cell growth appears in the control group, while QFF-PM_2.5_ exposure significantly decreases the cell proliferation along with the incubation time (*p* < 0.05 or 0.01). More specifically, 100 ng/mL QFF-PM_2.5_ exposure firstly attenuated the increase of cell density (*p* < 0.05) on day 6, and the significant inhibition in megakaryocytic proliferation was observed for all exposure groups (10, 50, 100 ng/mL) on day 12 (*p* < 0.01). The stagnation of megakaryocytic proliferation under these non-cytotoxic exposure levels could be correlated with the initiation of the cell differentiation [27]. Therefore, QFF-PM_2.5_ treatment at the non-cytotoxic level (≤ 0.1 μg/mL) for 12 days was ultimately chosen for the following thrombopoiesis studies.

### Thrombopoiesis from megakaryocytes induced by QFF-PM_2.5_

To further pinpoint whether QFF-PM_2.5_ exposure could cause the thrombopoiesis from megakaryocytes or not, the morphological observation was performed for the megakaryocytes from different groups. The result showed the typical small round cells in the control group, while the treatment of thrombopoietin (TPO, the positive control), a platelet growth factor, obviously enlarged cell volume (Fig. S3). Likely, QFF-PM_2.5_ treatment augmented the alteration of megakaryocytic morphology in a concentration-dependent manner (Fig. S4). The obvious changes in cell morphology suggested the initiation of megakaryocytic differentiation process.

**Fig. 2:**
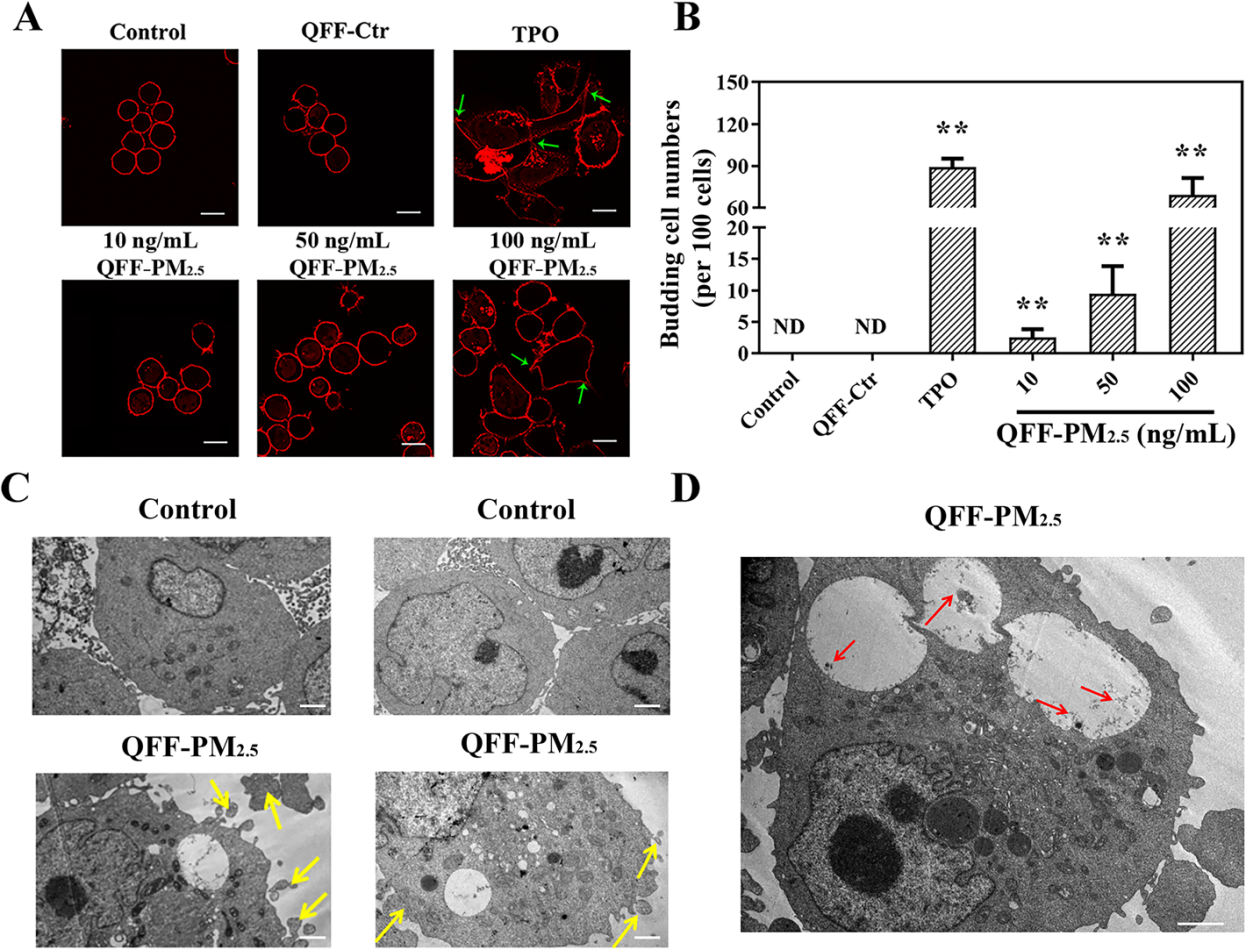
QFF-PM_2.5_ exposure promoted thrombopoiesis in megakaryocytes. **a** The fluorescence images of thrombopoiesis in megakaryocytes are characterized by immunostaining with wheat germ agglutinin (WGA, red fluorescence). The green arrows denote the budding cells. Scale bar = 15 μm. **b** Quantitative analysis of thrombopoiesis by counting the budding cell numbers in random views (*n* = 4). *p < 0.05, or **p < 0.01 versus the control. The blank filter extract (i.e. QFF-Ctr) and 1 ng/mL TPO were used as QFF-PM_2.5_ free and positive controls, respectively. **c**, **d** The representative TEM images of megakaryocytes. The yellow arrows refer to the features of mature megakaryocytes and thrombopoiesis, such as sponge-like edges, indicating the platelet demarcation membrane system. The red arrows indicate the fine particles in megakaryocytes. Scale bar = 2 μm. The exposure concentration of QFF-PM_2.5_ was 100 ng/mL, and the exposure lasted for 12 d

The impact of QFF-PM_2.5_ exposure on thrombopoiesis was further evaluated using wheat germ agglutinin (WGA) staining of cell membrane. The results in Fig. 2a show that all megakaryocytes in negative control have round contours, showing no detectable thrombopoiesis. The cells in the blank filter control (i.e. QFF-Ctr) had very similar morphology, confirming no spontaneous thrombopoiesis occurred. In contrast, the addition of TPO apparently changed the cell morphology, exhibiting irregular shapes and budding processes, suggesting the positive effect on megakaryocytic differentiation into platelets. As for the treatments of different concentrations of QFF-PM_2.5_, they also induced obvious morphological alterations in megakaryocytes, showing a similar induction effect on thrombopoiesis like that in TPO group. The quantitative analysis of thrombopoiesis by counting the budding cell numbers revealed TPO induced a significant increase in thrombopoiesis (*p* < 0.01, Fig. 2b). Likewise, an exposure concentration-dependent elevation in thrombopoiesis was also observed for QFF-PM_2.5_ treatments (Fig. 2b).

Transmission electronic microscopy (TEM) analysis illustrated that compared with the control, the cell volumes of megakaryocytes were significantly enlarged by QFF-PM_2.5_ exposure, and the cell margins were irregular and spongy, showing that QFF-PM_2.5_ promoted the thrombopoiesis from megakaryocytes (Fig. 2c). The yellow arrows indicated that QFF-PM_2.5_ treatment induced the appearance of the megakaryocytes with bud bulges, namely formation of precursor platelets (Fig. 2c). Additionally, the number of vesicles in megakaryocytes was increased, and some granular substances denoted by red arrows appeared in the vesicles (Fig. 2d). This finding suggested that fine particles could penetrate megakaryocytes via endocytosis and mainly concentrated in the vesicles. No obvious morphological alteration was observed for other organelles under TEM.

### QFF-PM_2.5_ elevated maturation and development of megakaryocytes

**Fig. 3:**
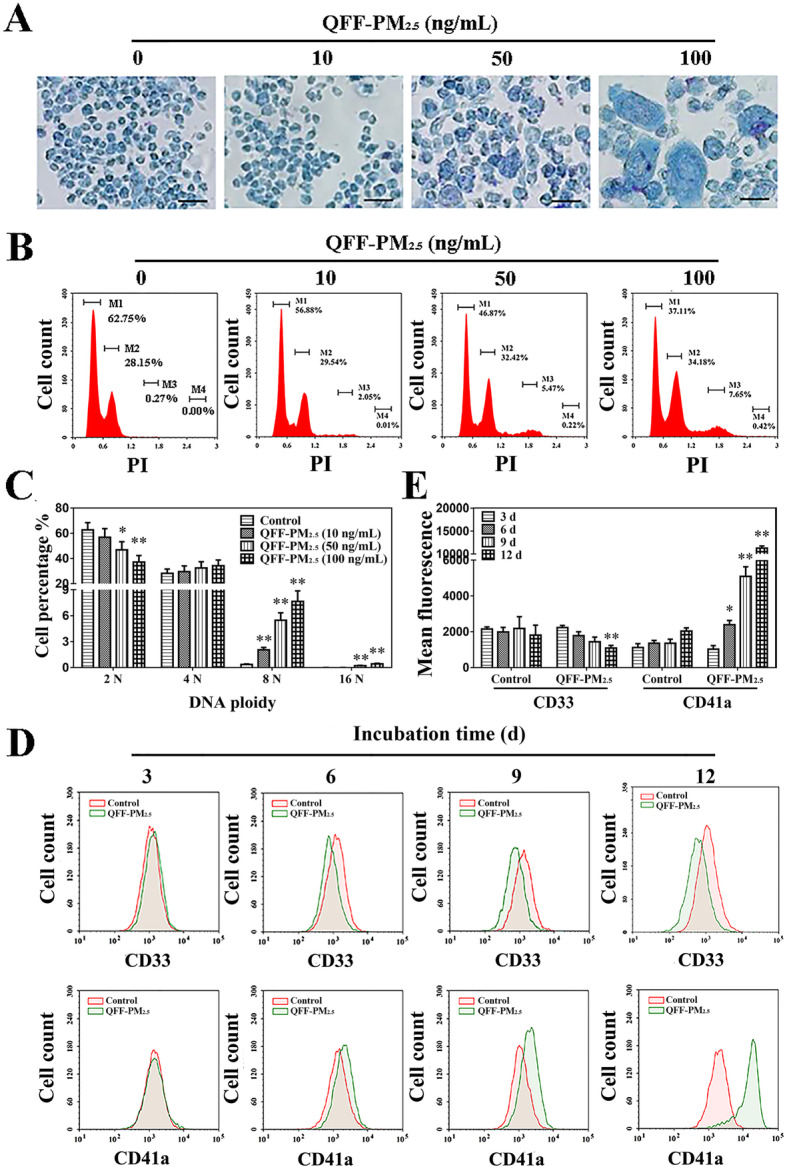
QFF-PM_2.5_ interfered with the maturation and development of megakaryocytes. **a** The morphological alteration of megakaryocyte using the Giemsa staining. Scale bar = 50 μm. **b** DNA ploidy in megakaryocytes using flow cytometry analysis. M1, M2, M3, and M4 refer to the 2 N, 4 N, 8 N, and 16 N ploidy, respectively. **c** The quantitative analysis of DNA ploidy in megakaryocytes (*n* = 4). **d** Time courses for the expressions of CD33 and CD41a in megakaryocytes. The exposure concentration of QFF-PM_2.5_ was 100 ng/mL. **e** The quantitative analysis of CD33 and CD41a expressions in megakaryocytes. *p < 0.05, and **p < 0.01 versus the corresponding controls

Given that megakaryocytes undergo a terminal maturation process to release platelets, [28] the potential influence of QFF-PM_2.5_ on the differentiation of megakaryocytes was further investigated to pinpoint the relevant mechanism of PM-promoted thrombopoiesis. The morphological observation of the megakaryocytes based on Giemsa staining assay showed that the treatment of positive control, TPO, enlarged the cell size and obviously changed the cell morphology (Fig. S5). In view of QFF-PM_2.5_ treatment, it enhanced the differentiation of megakaryocytes in a concentration-dependent manner (Fig. 3a).

The DNA contents evaluated using propidium iodide (PI) fluorescence intensity demonstrated TPO significantly induced elevation of DNA contents in megakaryocytes (Fig. S5), showing the promotion effect of this positive control on megakaryocytic differentiation. Likewise, QFF-PM_2.5_ treatment enhanced the contents of DNA in an exposure concentration-dependent manner (Fig. S6). Further DNA ploidy evaluation based on PI staining coupled with flow cytometry measurement showed that DNA ploidy was efficiently induced by QFF-PM_2.5_ exposure, as evidenced by the reduction of diploid cell population and the elevation of polyploid cell populations (Fig. 3b). The cellular DNA ploidy of 2 N changed significantly when the exposure concentration of QFF-PM_2.5_ was 50 ng/mL or higher, and significant increase in that of 8 N was accordingly induced by QFF-PM_2.5_ incubation in a dose-related manner (Fig. 3c, *p* < 0.05 or 0.01). These findings clearly verified that QFF-PM_2.5_ notably induced DNA ploidy in megakaryocytes, which was crucial during megakaryocytic maturation.

The characteristic molecular marker expression changes are commonly used to evaluate megakaryocytic differentiation and maturation [29]. The expressions of the cell surface myeloid antigen of CD33 and the megakaryocytic maturation associated antigen of CD41a were thus analyzed, and the results in Fig. S7 showed TPO obviously induced left shift of CD33 antibody-stained cell population peak and right shift of CD41a antibody-stained cell population peak, confirming the decrease of CD33 expression and increase of CD41a expression during the differentiation process of megakaryocytes. In view of airborne particles’ effect, the reduction in CD33 expression and the enhancement in CD41a expression were caused by 100 ng/mL QFF-PM_2.5_ exposure in time-dependent manners (Fig. 3d, e), showing the positive effect of QFF-PM_2.5_ on the development and maturation of megakaryocytes.

### Proteomic findings on QFF-PM_2.5_ induced megakaryocytic differentiation

**Fig. 4:**
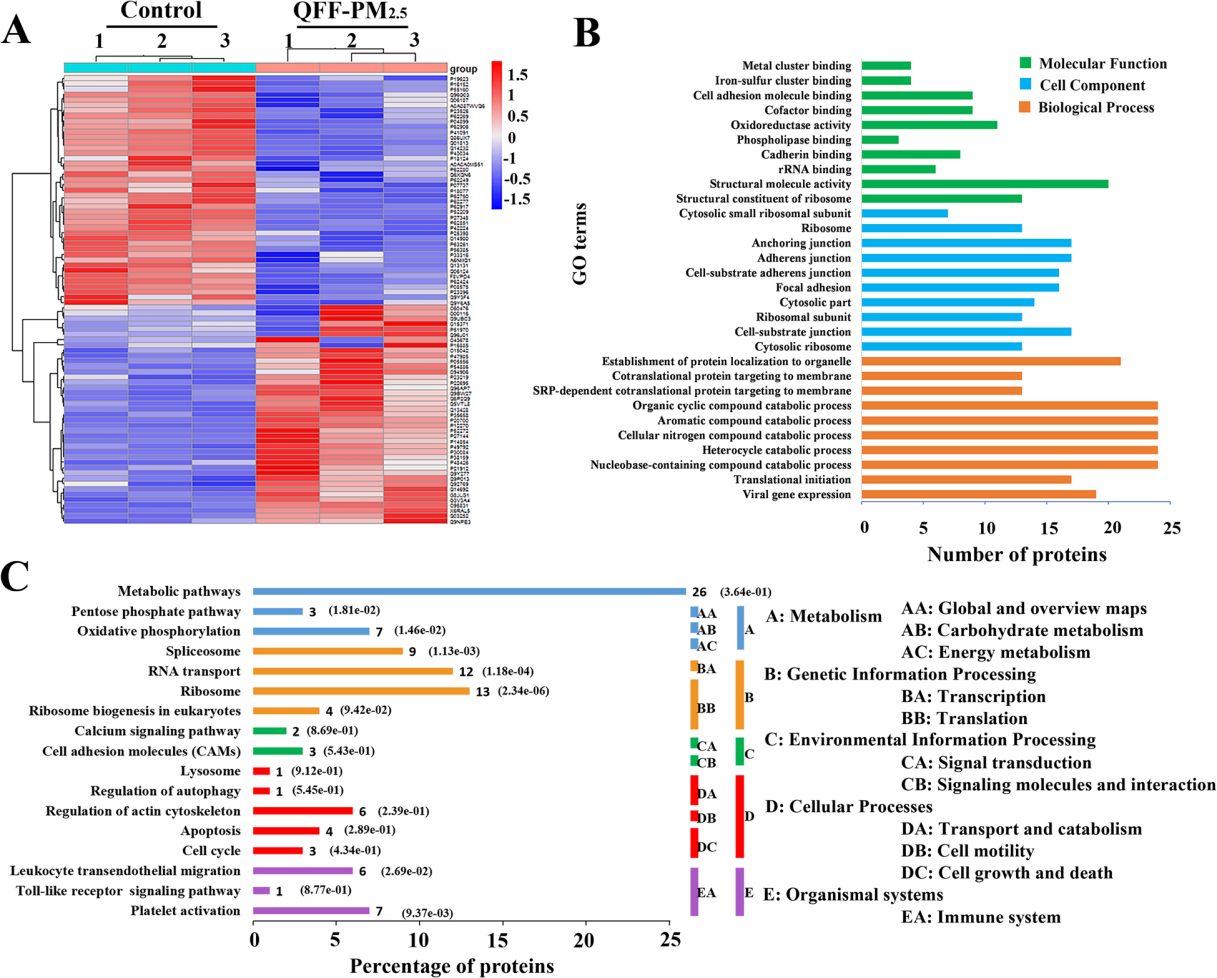
Proteomic analysis of megakaryocytes treated with QFF-PM_2.5_. **a** The clustering heatmap of differentially expressed proteins with |log_2_ ratio| ≥ 1 and *p*-value < 0.05. Each column represents an individual sample, and each row represents an individual protein. The samples were divided into two sub-groups including the control (nattier blue) and QFF-PM_2.5_ (pink) groups. **b** The three typically functional annotations, including biological process, molecular function, and cellular component, were classified by the gene ontology (GO) database. **c** Functional classification of differentially expressed proteins using the kyoto encyclopedia of genes and genomes (KEGG). The biological/metabolic annotation was given for each cluster of differentially expressed proteins

Differential proteomics analysis was performed to explore the crucial cellular and molecular events during megakaryocytic differentiation induced by QFF-PM_2.5_. As shown in the volcano plot (Fig. S8) the blue points indicated the differentially expressed proteins with large-magnitude fold-changes (|log_2_ ratio| ≥ 1, x-axis) and high statistical significance (*p* < 0.05, y-axis). More specifically, QFF-PM_2.5_ exposure upregulated the expressions of 337 proteins and downregulated the expressions of 358 proteins in megakaryocytes (Table S1). Extracting the target proteins correlated with the differentiation of megakaryocytes, a total of 84 differentially expressed proteins, including NADH dehydrogenase (O43678), cytochrome C oxidase subunit 6B1 (P14854), phosphatidylinositol 5-phosphate 4-kinase type-2 alpha (P48426), integrin beta-1 (P05556), and prostaglandin G/H synthase 1 (P23219), was obtained (Table S2) for the subsequent bioinformatics analysis. The heatmap (Fig. 4a) further illustrated the fold changes of these 84 differentially expressed proteins. Gene ontology (GO) enrichment analysis of the differentially-expressed proteins showed these differentially expressed proteins were involved in biological process, cell component and molecular function of megakaryocytes, and Fig. 4b shows the top 10 pathways involved in each category.

The differentially expressed proteins regulated by QFF-PM_2.5_ were further enriched in 17 kyoto encyclopedia of genes and genomes (KEGG) pathways (Fig. 4c), and protein-protein interaction network (PPI) (Fig. S9) was performed to reflect the correlation between the particular profile pattern and KEGG annotation. According to the KEGG annotation, the main pathways that regulate the differentiation of megakaryocytes caused by QFF-PM_2.5_ are concentrated in metabolic pathways, mitochondrial oxidative phosphorylation (OXPHOS), spliceosome, ribosome, platelet activation, etc. More specifically, a total of 6 differentially expressed proteins correlated with respiratory chain complex I-V, including NADH dehydrogenase (O43678), cytochrome C oxidase subunit 6B1 (P14854), mitochondrial cytochrome b-c1 complex subunit (P47985), NADH dehydrogenase [ubiquinone] 1 alpha subunit 8 (P51970), mitochondrial succinate dehydrogenase [ubiquinone] iron-sulfur subunit (P21912), and mitochondrial cytochrome b-c1 complex subunit 2 (P22695), were enriched in the OXPHOS pathway.

### Role of OXPHOS in QFF-PM_2.5_ influenced thrombopoiesis

**Fig. 5:**
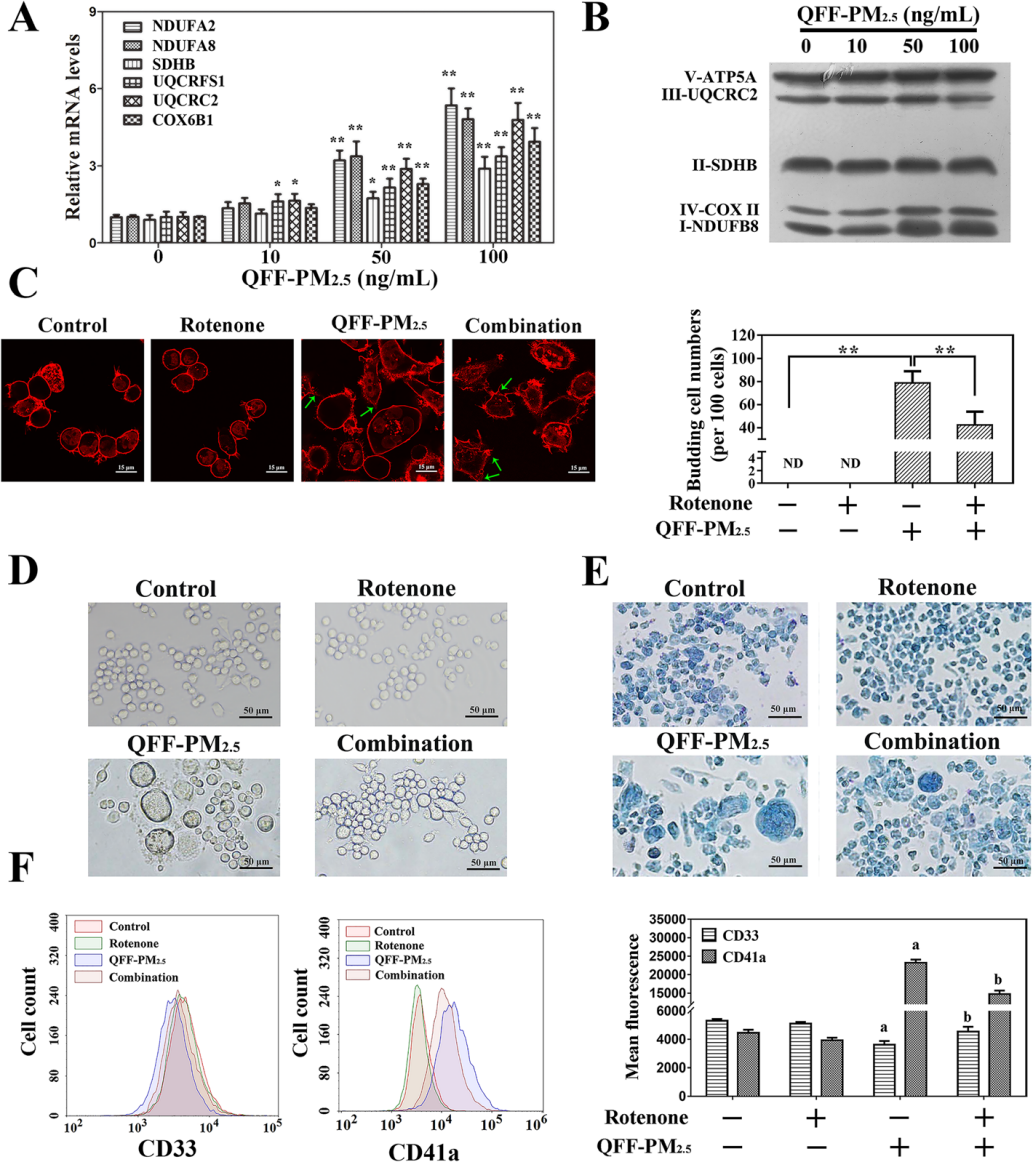
The role of mitochondrial OXPHOS on QFF-PM_2.5_ caused thrombopoiesis and megakaryocytic maturation. The effects of QFF-PM_2.5_ exposure on the mRNA levels (**a**) and protein expressions (**b**) of mitochondrial respiratory chain complex I-V in megakaryocytes. The antagonistic effects of rotenone on the thrombopoiesis (**c**) and morphological alterations in megakaryocytes induced by 100 ng/mL QFF-PM_2.5_ exposure based on bright field microscopic observations with or without Giemsa staining (**d**, **e**). **f** CD33 and CD41a levels in megakaryocytes upon 100 ng/mL QFF-PM_2.5_ treatment with or without 15 nM rotenone. **p < 0.01. ^a^*p* < 0.05 versus the negative controls, ^b^*p* < 0.05 versus the groups with QFF-PM_2.5_ treatment alone

To reveal the involvement of mitochondrial OXPHOS in QFF-PM_2.5_ disturbed differentiation of megakaryocytes, the transcriptional levels of mitochondrial respiratory chain complex were investigated, and the results in Fig. 5a show that QFF-PM_2.5_ induced exposure concentration-related increases in expressions of all test genes, including NDUFA2, NDUFA8, SDHB, UQCRFS1, UQCRC2 and COX6B1. This result was consistent with the finding from proteomics analysis. Western blot analysis for the protein biomarkers in mitochondrial OXPHOS illustrated that QFF-PM_2.5_ treatments enhanced the expressions of complex I (NDUFB8) and complex IV (COX II), while complex II (SDHB), complex III (UQCRC2) and complex V (ATP5A) remained unchanged (Fig. 5b and Fig. S10).

In order to further confirm the regulatory role of mitochondrial OXPHOS in QFF-PM_2.5_ induced thrombopoiesis from megakaryocytes, rotenone, an inhibitor of mitochondrial electron transport chain complex I, was used, and the addition of this inhibitor efficiently attenuated QFF-PM_2.5_ increased expressions of complex I and IV (Fig. S11). The morphological observation based on WGA staining showed that the co-treatment of rotenone with QFF-PM_2.5_ obviously reduced the counts of cells with irregular shapes and budding structures when compared with QFF-PM_2.5_ exposure group (Fig. 5c). This result was further confirmed by the findings from the bright field observation of the megakaryocytes from different treatments (Fig. 5d) and the images with Giemsa staining (Fig. 5e). The analysis of the biomarkers including CD33 and CD41a showed that the co-treatment of rotenone caused the antagonistic effects on QFF-PM_2.5_ induced alterations in the expressions of CD33 and CD41a in megakaryocytes (Fig. 5f). Accordingly, mitochondrial OXPHOS could play pivotal roles in QFF-PM_2.5_ elevated megakaryocytic maturation and thrombopoiesis.

Considering the disturbance of mitochondrial OXPHOS would influence reactive oxidative species (ROS) generation, and the mitochondrial function, like ROS generation, could play pivotal roles in regulating the differentiation of hematopoietic stem cells and megakaryocytes, [30–33] the effect of QFF-PM_2.5_ exposure on ROS levels in megakaryocytes was thus investigated. The results from flow cytometry assay using DCFH-DA probe showed that QFF-PM_2.5_ induced excessive ROS production in megakaryocytes (Fig. S12A), which was in an exposure concentration-dependent manner (Fig. S12B). The co-treatment of rotenone significantly attenuated the elevation in cellular ROS level induced by QFF-PM_2.5_ exposure (Fig. S12C, D). This result consistently confirmed oxidative stress could be induced by QFF-PM_2.5_ exposure through the regulation of mitochondrial OXPHOS in megakaryocytes, thus resulting in the accelerated thrombopoiesis.

### Evaluation of other airborne fine particles on thrombopoiesis

To investigate whether other airborne fine particle samples with different sizes collected by different filters had a similar effect with QFF-PM_2.5_, three other kinds of PM samples including PPF-PM_2.5_ (PM_2.5_ collected by organic polypropylene filter, i.e. PPF), QFF-PM_1_ (PM_1_ collected by QFF) and PPF-PM_1_, were tested. The characterization data in Fig. S13 show the particulate aggregates in morphology and hydrodynamic diameters at the nanometer scale. The data from chemical analysis was given in a previous publication [25]. The data from growth curves of megakaryocytes (Fig. S14) and WGA staining assay (Fig. S15A) demonstrated that QFF-PM_1_, PPF-PM_1_ and PPF-PM_2.5_ could also induce thrombopoiesis from megakaryocytes just like the effect of QFF-PM_2.5_. Using three-way ANOVA method, the statistical analysis of the influencing factors, including exposure concentration (EC), filter type (FT), PM size was performed for the quantitative data from Fig. 2b and S15B, and the result in Table S3-S5 shows that EC and PM size have significant effects on thrombopoiesis (*p* < 0.01), while FS has no statistically significant influence (*p* > 0.05).

The PI-probed DNA contents in megakaryocytes were also found to be increased by these three kinds of PM sample stimulations in exposure concentration-dependent manners (Fig. S16A). The DNA ploidy analysis using flow cytometry showed that it was induced by PM exposure, especially for 8 N ploidy, which exhibited in particle size dependent (Fig. S16B, C). The analysis of the megakaryocytic maturation biomarkers, i.e. CD33 and CD41a, showed QFF-PM_1_ also caused the decrease of CD33 expression and increase of CD41a expression (Fig. S17), which was consistent with the finding from QFF-PM_2.5_ exposure experiments (Fig. 3d, e). The statistical analysis for the effect of PM size on PM-influenced megakaryocytic maturation showed that this factor had significant effects on total DNA content and CD41a expression (Table S6), suggesting the role of PM size in disturbing thrombopoiesis.

## Discussion

The correlation between haze pollution and pulmonary or cardiovascular diseases has been well established by abundant epidemiological data. Platelet can be generated from megakaryocytes in lung, and regulate various physiological and pathological processes in circulative system, [10, 11] thus suggesting a potential target for airborne PM exposure in human bodies. The toxicological effect of PMs on thrombopoiesis from megakaryocytes is thus worthy of being explored to provide the pathological explanation for haze-induced health risks. In the present study, airborne fine particles significantly promoted the development, maturation and fragmentation of megakaryocytes, thus causing thrombopoiesis, and this process was regulated by mitochondrial oxidative phosphorylation (Fig. S18).

As far as PM-induced toxicity is concerned, the exposure dose selection is of utmost importance, taking into account that the toxicological outcomes could explain the chronic health risk in a realistic situation. Herein, the non-cytotoxic concentrations of PM no more than 100 ng/mL were selected to investigate the cellular responses of megakaryocytes under sub-lethal exposure stress of PMs. Considering the mean mass concentration of PM_2.5_ in Beijing, 2019 during the winter season was 210 μg/m^3^ (from 35 to 454 μg/m^3^), the daily inhaled PM amount was approximately 4.54 mg, taken the daily respiratory volume of 21.6 m^3^ for an adult [34]. When the PM bioavailability of 45% [29, 30] and pulmonary circulative blood of 100 mL [35–37] was assumed, the realistic exposure concentration could be 20.43 ng/mL, which indicated the study performed herein was environmental relevant. The findings accordingly could be used for assessing the actual haze-induced health risks.

The epidemiological data revealed the positive relationship between fine particle exposure and the increased number of platelets [16]. Previous animal experiments showed that the platelet count in the peripheral blood of mice exposed to 88.5 μg/m^3^ concentrated ambient particulate matters (CAPs) for 2 weeks was significantly increased as compared to the control [38]. The similar result that an elevated number of circulating platelets appeared in mice exposed to Sacramento urban PM_2.5_ for 24 h by Van et al. [39] The results in the present study indicated that the exposure of airborne fine particles promoted the thrombopoiesis from megakaryocytes, which showed the involvement of the differentiation of platelet progenitor cells, and provided the cytobiological explanation for PM-induced alteration of peripheral blood platelet number.

As a sound model alternative to human progenitor cells (CD34^+^) for studying thrombopoiesis, the megakaryocytic cells (Dami) proceed differentiation along with the growth of cell volume and nucleus enlargement due to the occurrence of DNA ploidy [19, 26]. In particular, megakaryocyte undergoes multiple DNA replications without cell divisions through a unique process of endomitosis [40]. With the occurrence of DNA polyploidy, the cells begin a rapid cytoplasmic expansion stage, which is characterized by the formation and accumulation of abundant proteins in cell membranes [40, 41]. As one of the characteristic molecular biomarkers, CD41a was upregulated during megakaryocytic maturation [42]. The cytoplasm of megakaryocytes subsequently undergoes large-scale recombination, forming beaded cytoplasmic extensions, i.e. pro-platelet [40]. The long branches of pro-platelets are then extended into sinusoidal vessels during cytoskeleton-driven processes, where they undergo fission to release platelet [41]. The results in Figs. 2 and 3 herein provided the supportive proof that airborne PM exposure interfered with the process of endomitosis and promoted the development of megakaryocytes. Likewise, Fortoul et al. found that the subacute and chronic inhalation of vanadium could induce the megakaryocytic maturation, and increase the cell size and cytoplasmic granular content in megakaryocytes, accompanying by nuclear changes with the final increase in circulating platelet production [43, 44]. Our finding was highly consistent with the report on fine particle-induced megakaryocytic mature and thrombopoiesis, and would indicate the potential deleterious risks from haze exposure under both healthy and disease conditions.

The mitochondrial OXPHOS plays important role in the hematopoiesis process [30, 31]. Recent researches have shown that mitochondrial activity or content determines the status of hematopoietic stem cells (HSCs), the source of the megakaryocytes, [32] and mitochondrial membrane potential (MPP) can influence the transcription rate of HSCs [45]. Quiescent and self-renewing HSCs rely on mitochondrial OXPHOS rather than on glycolysis for energy production, thus metabolically rewiring from glycolysis to mitochondrial-based energy generation accompanying with HSC differentiation and lineage commitment [31]. Additionally, excessive ROS generation is closely correlated with the dysfunction of mitochondrial OXPHOS and growing evidences support the crucial role of ROS in cellular responses to air pollution [46]. ROS has also been demonstrated to prime the differentiation of hematopoietic progenitors in Drosophila, [47] and the disturbance in oxidative/anti-oxidative system leads to premature HSC senescence due to loss of quiescence and self-renewal capacity in HSCs of mouse models [47, 48]. In the present study, the proteomic data revealed the disturbance in mitochondrial OXPHOS and elevation in cellular ROS level due to QFF-PM_2.5_ exposure (Figs. 4 and 5), confirming the disturbed mitochondrial OXPHOS and excessive cellular ROS production jointly regulated PM-induced megakaryocytic differentiation and thrombopoiesis.

The differentiation process of megakaryocytes, including cell maturation and thrombopoiesis, heavily rely on the recruitment of actin and microtubule cytoskeletons [49–51]. When encountering vascular damage, the recombination of cytoskeleton and doubling of actin filament content drive megakaryocytes to produce platelets [52]. The actin-binding proteins, such as filamin A (FlnA), also contribute to platelet production from megakaryocytes [53, 54]. Moreover, megakaryocytes express receptors to sense inflammation and IL-1 promotes the rupture of megakaryocytes and subsequent platelet release under acute inflammatory conditions [55–57]. The inflammatory responses to PM_2.5_ exposure are commonly believed to be related to the consequent health outcome [58]. The proteomic data herein showed the regulation of actin cytoskeleton (upregulation of PIP4K2A and ITGB1) and inflammatory responses (upregulation of PTGS1) due to QFF-PM_2.5_ exposure (Fig. 4, Table S2) could greatly contribute to its effect on megakaryocytic differentiation.

## Conclusion

In the present study, the effect of airborne fine particles on thrombopoiesis was explored, and the results demonstrated the induction effect of PMs on megakaryocytic development and maturation, which was fundamentally regulated by altering mitochondrial oxidative phosphorylation in cells. The findings, for the first time, provided the cytobiological basis for the cardiovascular health risk incurred by airborne particulate pollution. In future studies, more efforts should be devoted to the whole scale of the differentiation of HSCs into platelets, the function of these newly-formed platelets and in vivo biological responses to disclose the trajectory imprinted by haze exposure in human bodies.

## Materials and methods

### Cell culture and exposure protocol

Dami cell line (Institute of Biochemistry and Cell Biology, CAS, Shanghai, China), as a megakaryocytic cell model, was cultured in RPMI-1640 medium (HyClone, USA) supplemented with 10% fetal bovine serum (FBS, Gibico, USA) and 1% penicillin/streptomycin (Gibico, USA) in a humidified atmosphere of 5% CO_2_ at 37 °C.

In exposure experiments, the cells were seeded on the dishes or plates pre-coated with 1% gelatin (Gibico, USA) and cultured for 12 h, FBS concentration in the medium was adjusted from 10 to 2% before cell stimulation. The cells were treated with various concentrations of QFF-PM_2.5_ (0, 10, 20 and 100 ng/mL), and the exposure lasted for consecutive 12 d with the medium replacement every 3 d. Thrombopoietin (TPO, 1 ng/mL, Pepro Tech, USA) was used as the positive control to evaluate the differentiation of megakaryocytes into platelet. The blank control and the extract from the blank sampling filter (QFF-Ctr) were also designed as the negative controls.

### WGA staining assay

The megakaryocytic cells grown in 1% gelatin-precoated glass bottom cell culture dishes (35 mm, NEST) were performed 12 d exposure as described above. The culture medium was discarded, and the cells were gently washed with PBS twice, then fixed with 4% paraformaldehyde for 5 min at room temperature. After PBS rinse twice, the cells were incubated with 10 μg/mL WGA (Biotium, USA) at 37 °C for 30 min. Following triple washes with PBS, the samples from different groups were observed and photographed under a laser scanning confocal microscope (Leica TCS-SP5, Germany). The cells with morphological alterations were counted for the quantitative evaluation of thrombopoiesis.

### Ultrastructural analysis

The megakaryocytic cells from different exposure groups (around 5,000,000 megakaryocytic cells per group) were harvested, and immediately immersed into 2.5% glutaraldehyde (Sigma, USA) for 2 h, then washed with PBS buffer. The cells were post-fixed using 1% osmium tetroxide buffer (Sigma, USA) for 1 h, subsequently dehydrated using a series of concentrations of ethanol solutions, and finally embedded into epoxy resin. Sections were cut into 70 nm, and stained with 2% uranyl acetate (KEYI Technology Development Ltd., China) and 3% lead citrate (KEYI Technology Development Ltd., China) in sequence. The ultrastructural observation was performed under a transmission electron microscope (TEM, JEM 1200EX, Japan) with an accelerating voltage of 100 kV. The TEM images were analyzed under the help from the commercial-available technique service (Beijing ZKBC Technology Service Company Ltd).

### Cell morphological observation

In order to characterize the morphological changes during the differentiation of megakaryocytes, the cells were seeded in the 12-well plates at a density of 2000 cells/well to keep the initial confluency of about 5%. After the treatments with TPO (1 ng/mL, the positive control) or different concentrations of QFF-PM_2.5_ (0, 10, 50 and 100 ng/mL) for 12 d, the cells from different exposure groups were firstly observed under the inverted microscope (Olympus IX73, Japan) and the images under the bright field were taken. Then the cells were gently washed twice with PBS, fixed with 1 mL of cold methanol (− 20 °C) for 3 min, and stained with 10% Giemsa (Solarbio, China) at room temperature for 20 min. The stained cells were washed with PBS again and finally submitted to morphological observation and photographing.

### DNA polyploidy analysis

The megakaryocytic cells were seeded in 1% gelatin pre-coated 60 mm dishes at the density of 100,000 cells/dish, and performed exposure experiments as described above. When the exposure was terminated, the cells were washed with PBS twice, harvested, fixed in ice-cold 70% ethanol and kept at − 20 °C for 24 h. After rinsing with PBS twice using centrifugation (600 g, 5 min), the cells were incubated with 0.1 mg/mL RNase A (Solarbio, China) at 37 °C for 30 min, and stained with 10 μg/mL propidium iodide (PI, Solarbio, China) in the dark at room temperature for 10 min. The cells were subsequently washed twice with PBS, dispersed in 500 μL of PBS, and finally analyzed using a flow cytometer (Novocyte 1040, ACEA Biosciences, USA) under λ_ex_/λ_em_ of 535 nm/615 nm for DNA polyploidy. The percentages of cell populations with DNA ploidy of 2 N, 4 N, 8 N and 16 N were evaluated using Novo Express software.

### Flow cytometry analysis of CD33 and CD41a expressions in megakaryocytes

The megakaryocytes were seeded in 1% gelatin pre-coated 60 mm dishes at the density of 100,000 cells/dish and processed stimulation with 100 ng/mL QFF-PM_2.5_ for 3, 6, 9 and 12 d. The cells harvested at different time points were washed once with ice-cold PBS and subsequently incubated with 100 μL of CD33-PE (1:10, BioLegend, USA) and CD41a-FITC (1:10, Invitrogen, USA) in the dark at 37 °C for 30 min, respectively. Followed by the centrifugation (7000 rpm, 30 s) and three-time washes with PBS, the cells were re-suspended in 500 μL of PBS, and submitted to the analysis by a flow cytometer (Novocyte 1040, ACEA Biosciences, USA). CD41a expression was assessed using the fluorescein isothiocyanate (FITC) channel, whereas CD33 level was examined using the PE channel. The forward and side scatters were used to eliminate the disturbance from cellular fragments. The quantitation of the test biomarker expressions was based on the corresponding mean fluorescence.

### Differential proteomics analysis

The megakaryocytic cells were seeded in 1% gelatin pre-coated 100 mm dishes at the density of 1,000,000 cells/dish, cultured for 12 h and performed exposure experiments as described above. Three biological replicates from the control group and 100 ng/mL QFF-PM_2.5_ exposure group (12 d), were prepared for proteomic analysis. Briefly, 1 × 10^7^ cells were harvested and lysed with 300 μL of SDT lysis buffer (4% SDS, 100 mM DTT, 100 mM Tris-HCl pH 8.0). Samples were boiled for 5 min and further ultrasonicated and boiled again for another 5 min. Followed by the centrifugation at 16,000 g for 15 min to remove undissolved cellular debris, the supernatant was collected and quantified with a BCA protein assay kit (Bio-Rad, USA). Extracted proteins were separated by 12.5% SDS-PAGE gel, and protein bands were visualized by Coomassie Blue R-250 staining. Protein digestion in gel pieces was performed with a filter aided proteome preparation (FASP) protocol as previously described for proteins digestion [59].

Liquid chromatography (LC) was performed on a Q Exactive Plus mass spectrometer (MS) coupled with Easy 1200 nLC (Thermo Fisher Scientific, MA, USA). Reverse-phase high-performance liquid chromatography (RP-HPLC) separation was performed by a self-packed column (75 μm × 150 mm; 3 μm ReproSil-Pur C18 beads, 120 Å, Dr. Maisch GmbH, Ammerbuch, Germany) at a flow rate of 300 nL/min. The RP-HPLC mobile phase A was 0.1% formic acid in the water, and B was 0.1% formic acid in 85% acetonitrile. The gradient elution was set as following: 2–8% buffer B (2 min), 8 to 28% buffer B (40 min), 28 to 40% buffer B (8 min), 28 to 40% buffer B (1 min), and 40 to 100% buffer B (10 min).

The MS data, analyzed using MaxQuant software, were searched against the Uniprot protein *Homo sapiens* database (174,301 total entries, downloaded 09/17/2018). Label-free quantification was carried out using intensity determination and normalization algorithm as previously described [60]. The bioinformatics data were processed using Perseus software, Microsoft Excel, R statistical computing software and Cytoscape software.

### Transcriptional assay for mitochondrial respiratory chain genes

The megakaryocytic cell samples from different treatments were homogenized using Trizol reagent (Gibco, USA) for RNA extraction and purification according to the manufacturer’s instruction. The RNA concentrations were measured by NanoDrop (Thermo Scientific, USA). After the transcription of RNA to cDNA using the script cDNA Synthesis kit (BioRad, USA), all the samples were performed for *q*PCR assay using SYBR Green *q*PCR Master Mix (BioRad, USA) in a Roche 480 Real-Time PCR system (Roche, USA). The target genes included ubiquinone oxidoreductase subunit A2 (NDUFA2), ubiquinone oxidoreductase subunit A8 (NDUFA8), succinate dehydrogenase complex iron-sulfur subunit B (SDHB), ubiquinol-cytochrome c reductase, Rieske iron-sulfur polypeptide 1 (UQCRFS1), ubiquinol-cytochrome c reductase core protein 2 (UQCRC2), cytochrome c oxidase subunit 6B1 (COX6B1) and the reference gene was GAPDH. The corresponding primer sequences are shown in Table S7. The relative mRNA expressions of target genes were normalized to the housekeeping gene using 2^–ΔΔCT^ method [61].

### Immunoblotting assay for respiratory chain complex

The megakaryocytes from different treatments (0, 10, 50 and 100 ng/mL, 12 d) were transferred to a glass homogenizer and ground in an ice bath for 35 min. Then, the mitochondrial proteins were prepared using a mitochondrial protein extraction kit according to the manufacturer’s instruction (Nanjing Jiancheng Bioengineering Institute, Nanjing, China). After being quantitatively analyzed using a BCA protein assay kit (Beyotime, China), the consistent amount of the protein sample from each group was submitted to Western blot assay. The primary antibody was the total OXPHOS antibody cocktail (1:1000, ab110411, Abcam, USA), and the second antibody was the HRP-conjugated anti-mouse IgG (1:500, EasyBio, China). The quality of this assay was reversely confirmed by stable expressions of the test biomarkers, including mitochondrial respiratory chain complex II, III and V.

### Assays for co-treatment of PMs and rotenone

To further analyze the regulatory role of mitochondrial OXPHOS in PM-induced differentiation of megakaryocytes into platelets, rotenone, an inhibitor of complex I of the mitochondrial electron transport chain, was used for the co-exposure experiments. Briefly, four groups including control, 15 nM rotenone, 100 ng/mL QFF-PM_2.5_ and the combination of rotenone (15 nM) and QFF-PM_2.5_ (100 ng/mL) were designed, and the exposure lasted for 12 d as described above. After the treatment was terminated, WGA staining, morphological observation and flow cytometry analysis of CD33 and CD41a expressions were performed to evaluate the effect of rotenone on PM-induced thrombopoiesis.

### Statistical analysis

All statistical analyses were performed using the software of SPSS 17.0 or GraphPad Prism 8.0.1. Each assay was independently carried out three times or more, and the quantitative data were expressed as the mean ± standard deviation (SD), unless indicated otherwise. Differences among groups were evaluated by one-way analysis of variance (ANOVA) or three-way ANOVA. Statistical significance was denoted when *p* value was less than 0.05, or 0.01.

## Supplementary Information

**Additional file 1: Figure S1.** The effect of QFF-PM_2.5_ exposure on cell viabilities of megakaryocytes. **Figure S2.** Growth curves of megakaryocytes upon QFF-PM_2.5_ exposure. **Figure S3.** The morphological alteration of megakaryocytes upon TPO treatment. **Figure S4.** The morphological alteration of megakaryocytes upon QFF-PM_2.5_ treatment. **Figure S5.** Giemsa staining assay for TPO-induced morphological alteration in megakaryocytes. **Figure S6.** The contents of DNA in megakaryocytes upon QFF-PM_2.5_ treatment. **Figure S7.** The levels of CD33 and CD41a in megakaryocytes are stimulated by TPO. **Figure S8.** Volcano plot for the distribution of differentially expressed proteins. **Figure S9.** The crosstalk of critical differentially-expressed proteins involved pathways using protein-protein interaction network (PPI). **Figure S10.** The effects of QFF-PM_2.5_ exposure on the protein expressions of mitochondrial respiratory chain complex I-V in megakaryocytes. **Figure S11.** The antagonistic effect of rotenone on QFF-PM_2.5_ influenced mitochondrial oxidative phosphorylation in megakaryocytes. **Figure S12.** QFF-PM_2.5_ induced mitochondrial ROS generation. **Figure S13.** Characterization of airborne fine particles. **Figure S14.** Growth curves of megakaryocytes in different PM exposure groups. **Figure S15.** The effects of different PM samples on thrombopoiesis from megakaryocytes. **Figure S16.** The effects of different PMs on DNA ploidy in megakaryocytes. **Figure S17.** The effect of QFF-PM_1_ treatment on expressions of CD33 and CD41a in megakaryocytes. **Figure S18.** The illustration of thrombopoiesis upon PM exposure and the underlying mechanism. **Table S1.** The numbers of differentially expressed proteins in megakaryocytes (*p* value < 0.05 and |log_2_ ratio| ≥ 1). **Table S2.** The differentially expressed proteins correlated with megakaryocyte differentiation. **Table S3.** Statistical analysis of the influencing factors for PM-induced thrombopoiesis. **Table S4.** The influence of PM sampling filter type on thrombopoiesis. **Table S5.** The influence of PM size on thrombopoiesis. **Table S6.** Statistical analysis for the effect of PM size on PM-promoted megakaryocytic maturation. **Table S7.** Pair primer sequences for quantitative polymerase chain reaction (*q*PCR) analysis.

## Data Availability

All data analyzed within this study are included either in the manuscript or in the additional supplementary files.

## Abbreviations

PM: Particulate matter
OXPHOS: Oxidative phosphorylation
MEP: Megakaryocyte/erythrocyte progenitor
HSC: Hematopoietic stem cells
QFF: Quartz fiber filter
PPF: Polypropylene filter
TEM: Transmission electron microscopy
WGA: Wheat germ agglutinin
TPO: Platelet growth factor
PI: Propidium iodide
GO: Gene ontology
KEGG: Kyoto encyclopedia of genes and genomes
PPI: Protein-protein interaction network
CAPs: Concentrated ambient particulate matter
MPP: Mitochondrial membrane potential
ROS: Reactive oxygen species
FBS: Fetal bovine serum
WGA: Wheat germ agglutinin
