# Supplementary material for: Airborne particulate matters induce thrombopoiesis from megakaryocytes through regulating mitochondrial oxidative phosphorylation
Source: Part Fibre Toxicol. 2021 May 13;18:19. doi: 10.1186/s12989-021-00411-4 (PMC8117637; doi:10.1186/s12989-021-00411-4)
Supplement: Supplementary file 1 — Additional file 1: Figure S1. The effect of QFF-PM2.5 exposure on cell viabilities of megakaryocytes. Figure S2. Growth curves of megakaryocytes upon QFF-PM2.5 exposure. Figure S3. The morphological alteration of megakaryocytes upon TPO treatment. Figure S4. The morphological alteration of megakaryocytes upon QFF-PM2.5 treatment. Figure S5. Giemsa staining assay for TPO-induced morphological alteration in megakaryocytes. Figure S6. The contents of DNA in megakaryocytes upon QFF-PM2.5 treatment. Figure S7. The levels of CD33 and CD41a in megakaryocytes are stimulated by TPO. Figure S8. Volcano plot for the distribution of differentially expressed proteins. Figure S9. The crosstalk of critical differentially-expressed proteins involved pathways using protein-protein interaction network (PPI). Figure S10. The effects of QFF-PM2.5 exposure on the protein expressions of mitochondrial respiratory chain complex I-V in megakaryocytes. Figure S11. The antagonistic effect of rotenone on QFF-PM2.5 influenced mitochondrial oxidative phosphorylation in megakaryocytes. Figure S12. QFF-PM2.5 induced mitochondrial ROS generation. Figure S13. Characterization of airborne fine particles. Figure S14. Growth curves of megakaryocytes in different PM exposure groups. Figure S15. The effects of different PM samples on thrombopoiesis from megakaryocytes. Figure S16. The effects of different PMs on DNA ploidy in megakaryocytes. Figure S17. The effect of QFF-PM1 treatment on expressions of CD33 and CD41a in megakaryocytes. Figure S18. The illustration of thrombopoiesis upon PM exposure and the underlying mechanism. Table S1. The numbers of differentially expressed proteins in megakaryocytes (p value < 0.05 and |log2 ratio| ≥ 1). Table S2. The differentially expressed proteins correlated with megakaryocyte differentiation. Table S3. Statistical analysis of the influencing factors for PM-induced thrombopoiesis. Table S4. The influence of PM sampling filter type on thrombopoiesis. Table S5. The [file 12989_2021_411_MOESM1_ESM.docx]

**Supplementary Material**

**Airborne particulate matters induce thrombopoiesis from megakaryocytes through regulating mitochondrial oxidative phosphorylation**

Xiaoting Jin^1,2^, Hongyan Yu^2^, Baoqiang Wang^2^, Zhendong Sun^1,3^, Ze Zhang^2^, Qian S Liu^1^, Yuxin Zheng^2^, Qunfang Zhou^1,3,4,5,*^, and Guibin Jiang^1,3,4^

^1^State Key Laboratory of Environmental Chemistry and Ecotoxicology, Research Center for Eco-Environmental Sciences, Chinese Academy of Sciences, Beijing, 100085, P. R. China.

^2^China School of Public Health, Qingdao University, Qingdao 266071, P. R. China.

^3^School of Environment, Hangzhou Institute for Advanced Study, University of Chinese Academy of Sciences, Hangzhou, 310000, P. R. China.

^4^College of Resources and Environment, University of Chinese Academy of Sciences, Beijing, 100049, P. R. China.

^5^Institute of Environment and Health, Jianghan University, Wuhan 430056, P. R. China.

* Corresponding Author:

Dr. Qunfang Zhou, State Key Laboratory of Environmental Chemistry and Ecotoxicology, Research Center for Eco-Environmental Sciences, Chinese Academy of Sciences, Beijing 100085, P. R. China.

Email: zhouqf@rcees.ac.cn

### Supplementary materials and methods

**Sampling and characterization of airborne fine particles**

The airborne fine particulate matter (PM) including PM_1_ and PM_2.5_ were collected using an inorganic quartz filter (QFF) and organic polypropylene filter (PPF) during the heating season in Beijing. The details about the sampling process and PM preparation were provided in a previous study.[1] The morphologies of four kinds of PM samples (i.e. QFF-PM_2.5_, QFF-PM_1_, PPF-PM_2.5_, and PPF-PM_1_) were analyzed by transmission electron microscopy (TEM) (JEOL, 2100F, Japan). The hydrodynamic diameters of all fine particles suspended in Milli-Q water were evaluated by a Zeta Sizer Nano ZS (Malvern Nano ZS, Nalvem, UK).

**Alamar Blue assay**

To screen the non-cytotoxic exposure levels of QFF-PM_2.5_, the megakaryocytic cells with the density of 10,000 cells/well were seeded in the 96-well plates and cultured for 12 h. The cells were subsequently treated with 0, 0.05, 0.1, 0.5, 1, 5, 10 and 50 μg/mL PMs for 48 hours (h). When the exposure procedure was terminated, the cells were observed and photographed using an inverted microscope (Olympus IX73, Japan). In each well, 10 μL of 100 μM celltiter-blue reagent (Sigma Aldrich, USA) was added to each well, mixed and incubated for another 2 h, and the fluorescence was recorded at 530 nm/590 nm (λ_ex_/λ_em_) using a microplate reader (Thermo Fisher Scientific, USA).

To analyze the growth of megakaryocytic cells under the exposure stress of QFF-PM_2.5_, the cells were plated on the 96-well plates at the density of 2000 cells/well. After 12-h incubation, the cell medium was replaced by 100 μL of cell medium containing 5% FBS, and the cells were treated with various concentrations of airborne fine particles (0, 10, 50 and 100 ng/mL) for 0, 3, 6, 9, and 12 d, respectively, and six parallel wells were set for each condition. The cell viability was measured using the alamarBlue assay described above.

**Assay for ROS level**

The megakaryocytic cells were seeded in 6-well plates at the densities of 30,000 cells/well, and treated with 10, 50, and 100 ng/mL QFF-PM_2.5_ for 12 d. The negative control and QFF-ctr groups were designed in parallel. After exposure, the cells were digested by trypsin, centrifuged at 1200 rpm for 5 min, suspended in 1 mL of serum-free medium containing 10 μM DCFH-DA (Invitrogen, USA), incubated in the dark for 20 min under a gentle upside down mixing at 5 min intervals. The cells were subsequently washed with serum-free medium twice, resuspended in 500 μL of PBS, and submitted to the analysis of intracellular ROS using a flow cytometer (Novocyte 1040, ACEA Biosciences, USA) under λ_ex_/λ_em_ of 488 nm/525 nm.

**Tests for the effects of QFF-PM_1_, PPF-PM_2.5_, and PPF-PM_1_**

The megakaryocytic cells were treated with a series of concentrations of PPF-PM_2.5_, QFF-PM_1_ and PPF-PM_1_ (10, 50, and 100 ng/mL) for consecutive 12 d and submitted to the assays including WGA staining, DNA ploidy, CD33 and CD41a expressions following the similar protocols to those used for QFF-PM_2.5_.


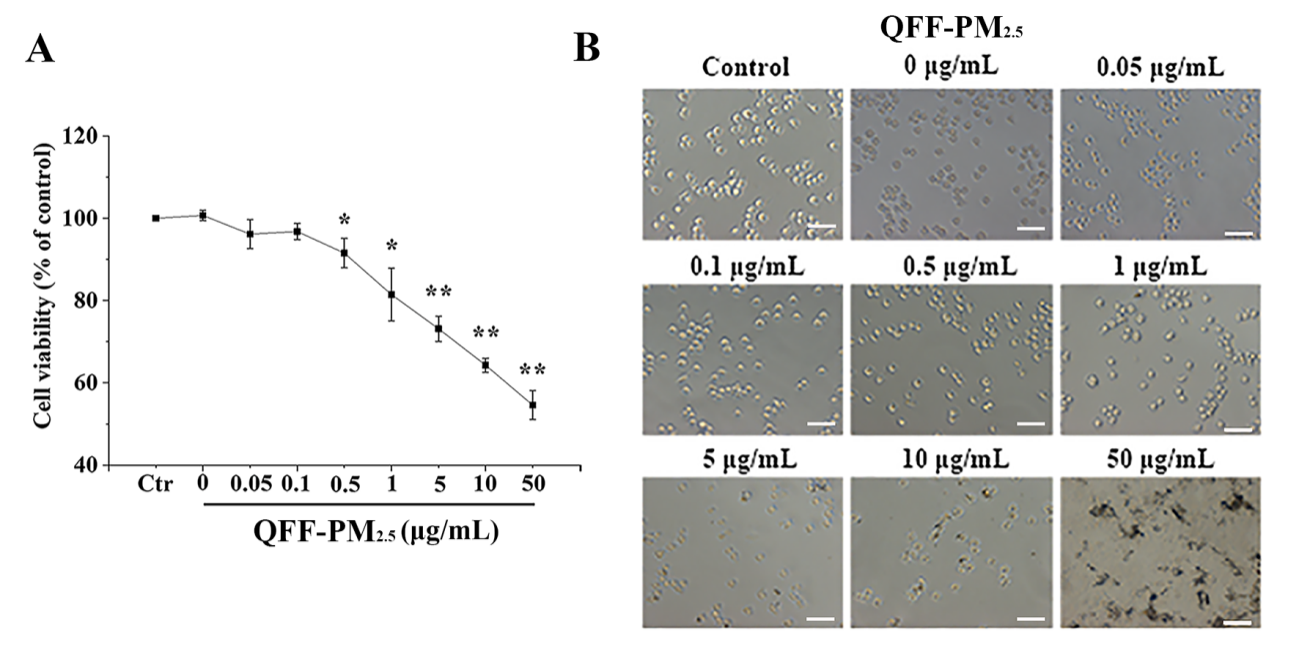


**Fig. S1.** The effect of QFF-PM_2.5_ exposure on cell viabilities of megakaryocytes. (A) The relative cell viabilities of megakaryocytes under a series of concentrations of QFF-PM_2.5_ treatment. **p* < 0.05, or ***p* < 0.01 versus the negative control. (B) The morphological alterations of megakaryocytes caused by QFF-PM_2.5_ stimulation. The exposure time lasted for 48 h. The scale bar represents 100 μm.


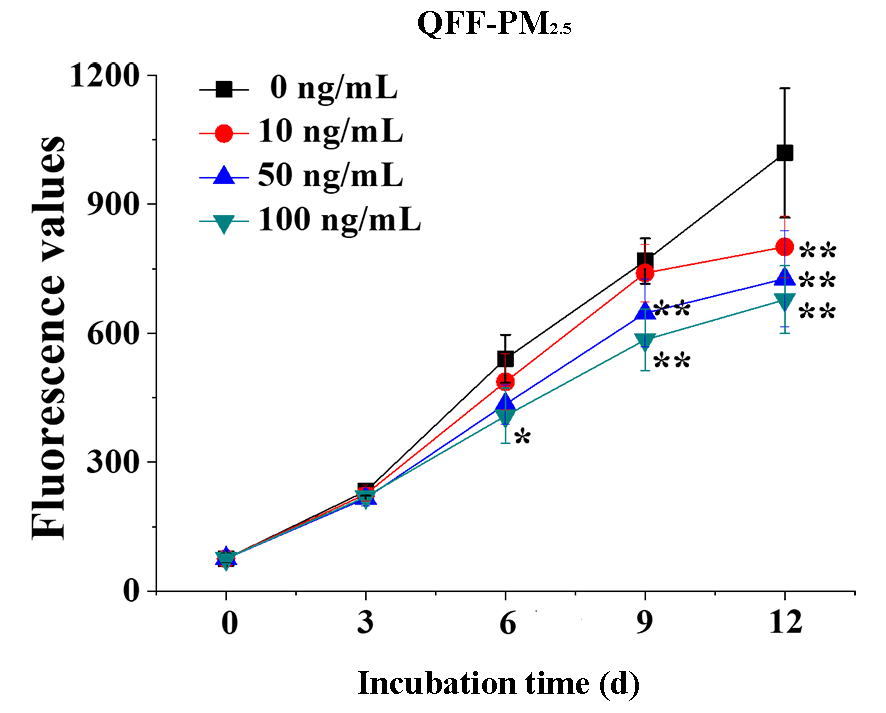


**Fig. S2.** Growth curves of megakaryocytes upon QFF-PM_2.5_ exposure. **p* < 0.05, or ***p* < 0.01 versus the control group.


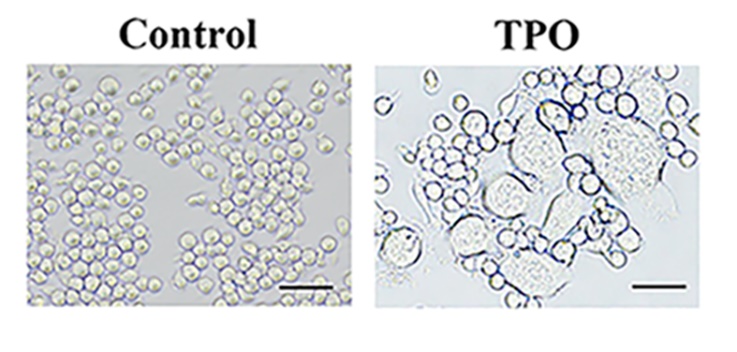


**Fig. S3.** The morphological alteration of megakaryocytes upon TPO treatment. The concentration of the positive control (TPO) is 1 ng/mL. Scale bars equal to 50 μm.


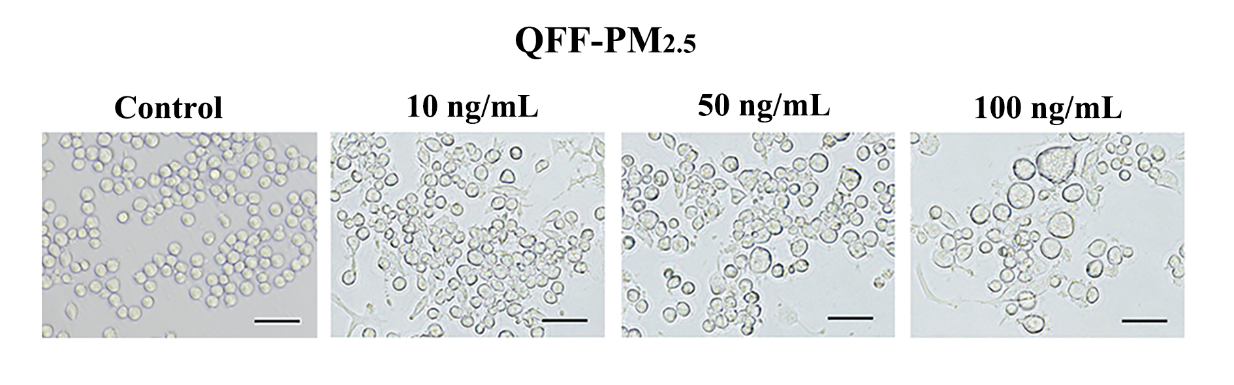


**Fig. S4.** The morphological alteration of megakaryocytes upon QFF-PM_2.5_ treatment. Scale bars equal to 50 μm.


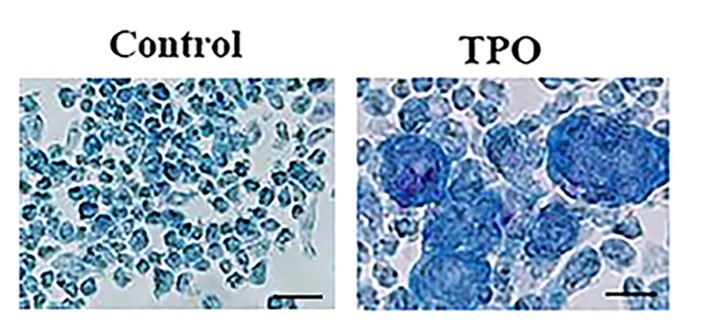


**Fig. S5.** Giemsa staining assay for TPO-induced morphological alteration in megakaryocytes. Scale bars equal to 50 μm.


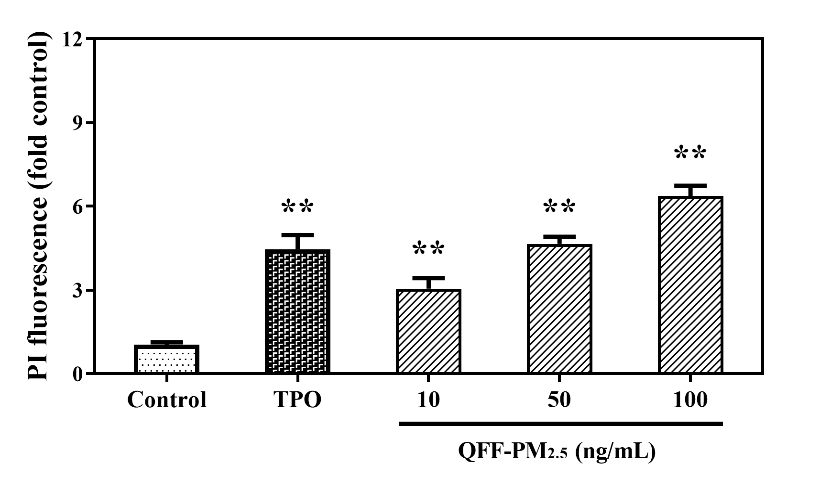


**Fig. S6.** The contents of DNA in megakaryocytes upon QFF-PM_2.5_ treatment. TPO (1 ng/mL) was used as a positive control. **p* < 0.05, or ***p* < 0.01 versus the control group.


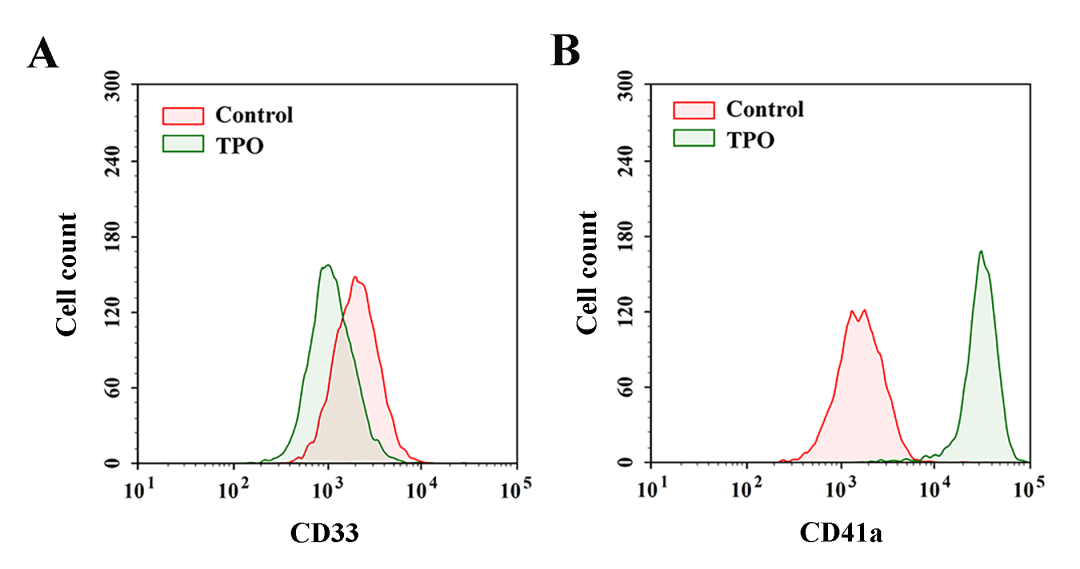


**Fig. S7.** The levels of CD33 and CD41a in megakaryocytes are stimulated by TPO. (A) CD33-expressed cell population. (B) CD41a-expressed cell population. The exposure was performed using 1 ng/mL TPO for 12 d.


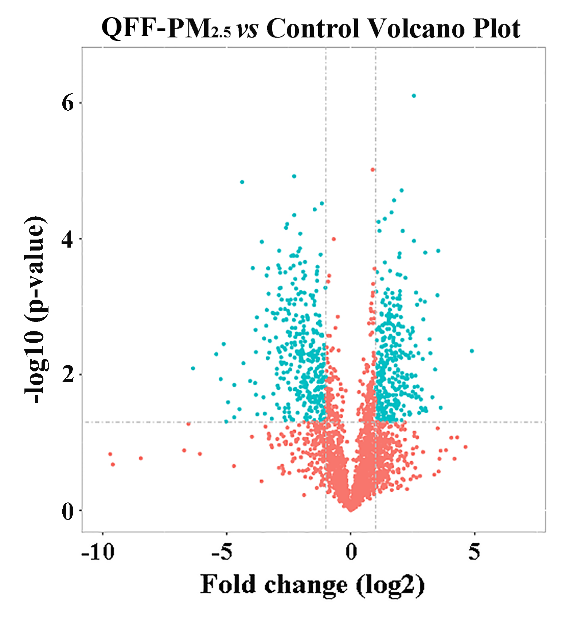


**Fig. S8.** Volcano plot for the distribution of differentially expressed proteins. Each point represented on protein, and those in blue indicated the proteins that displayed both |log_2_ ratio| ≥ 1 and *p*-value < 0.05.


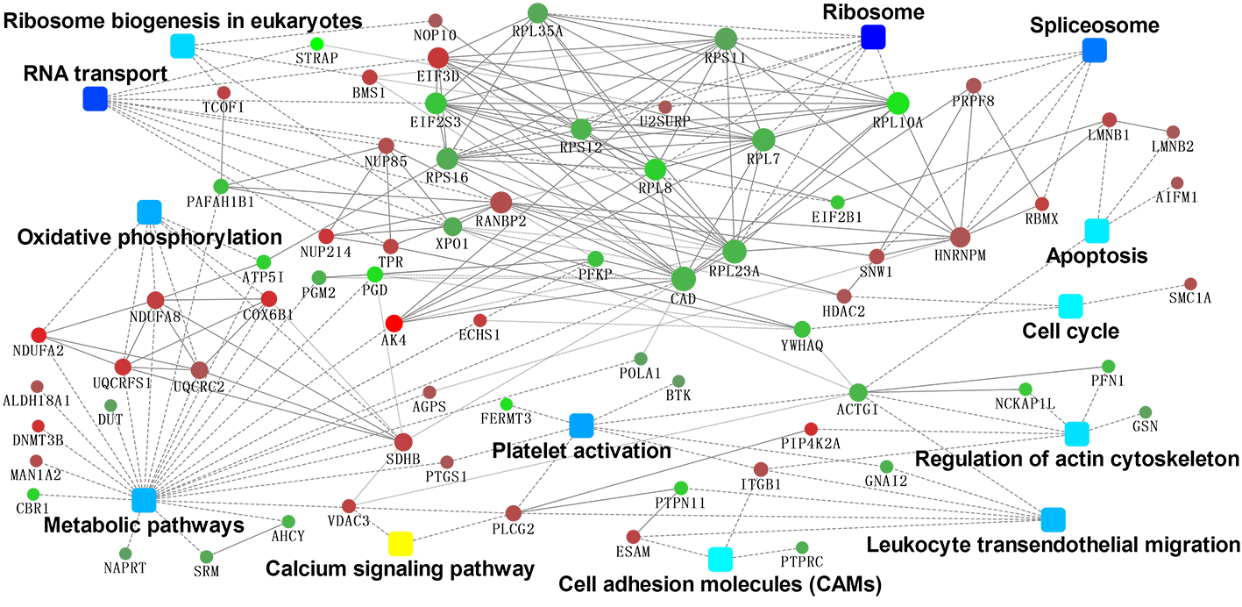


**Fig. S9.** The crosstalk of critical differentially-expressed proteins involved pathways using protein-protein interaction network (PPI). The proteins (indicated by circles) are enriched in the pathways (indicated by squares). Each protein profile is represented in a different color.


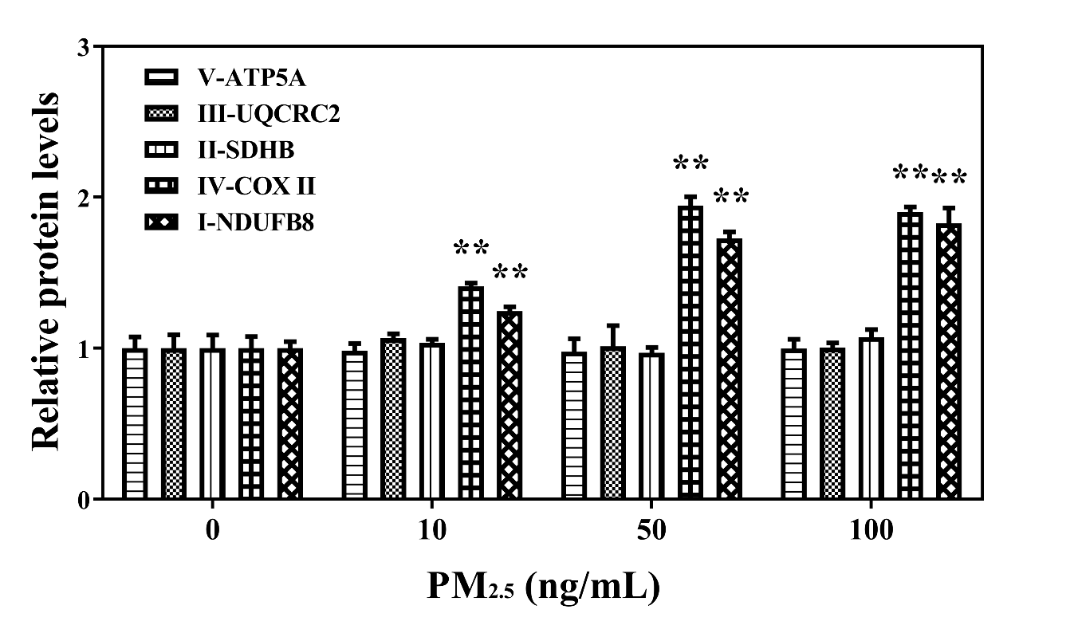


**Fig. S10.** The effects of QFF-PM_2.5_ exposure on the protein expressions of mitochondrial respiratory chain complex I-V in megakaryocytes. ***p* < 0.01 versus the controls.


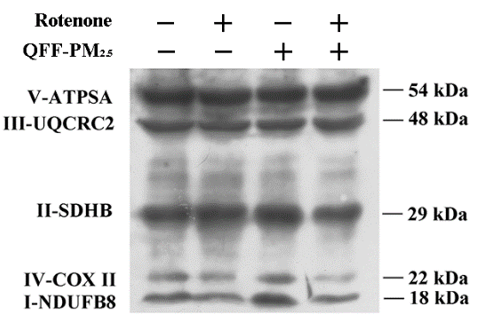


**Fig. S11.** The antagonistic effect of rotenone on QFF-PM_2.5_ influenced mitochondrial oxidative phosphorylation in megakaryocytes. The exposure concentrations of QFF-PM_2.5_ and rotenone were 100 ng/mL and 15 nM, respectively, and the exposure lasted for 12 d.


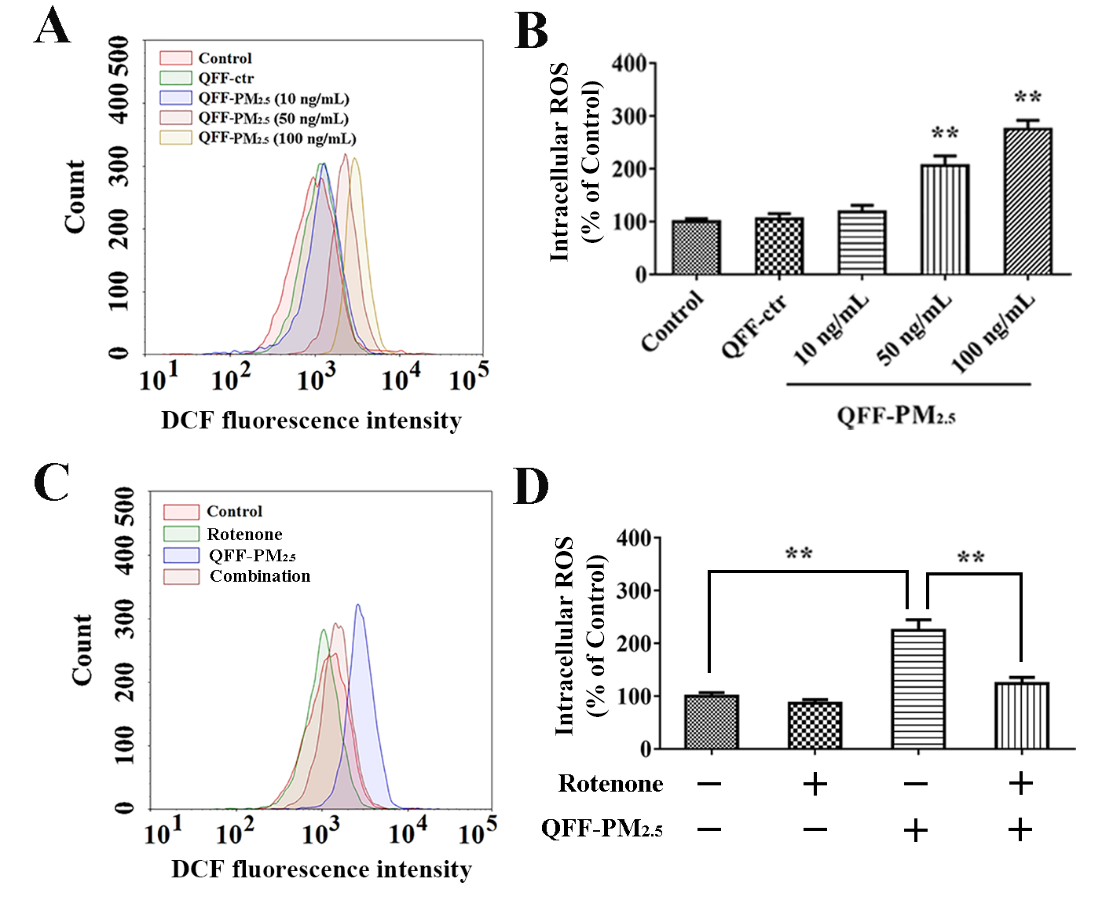


**Fig. S12.** QFF-PM_2.5_ induced ROS generation. (A) Intracellular ROS generation using flow cytometry analysis. (B) Quantitative analysis of ROS generation in different treatments. ***p* < 0.01 versus QFF-ctr control. (C) The effect of rotenone on QFF-PM_2.5_ induced ROS generation in megakaryocytes. (D) Quantitative analysis for the inhibition effect of rotenone on QFF-PM_2.5_ induced ROS generation. The exposure concentrations of QFF-PM_2.5_ and rotenone were 100 ng/mL and 15 nM, respectively, and the exposure lasted for 12 d. ***p* < 0.01 versus the control or QFF-PM_2.5_ exposure groups.


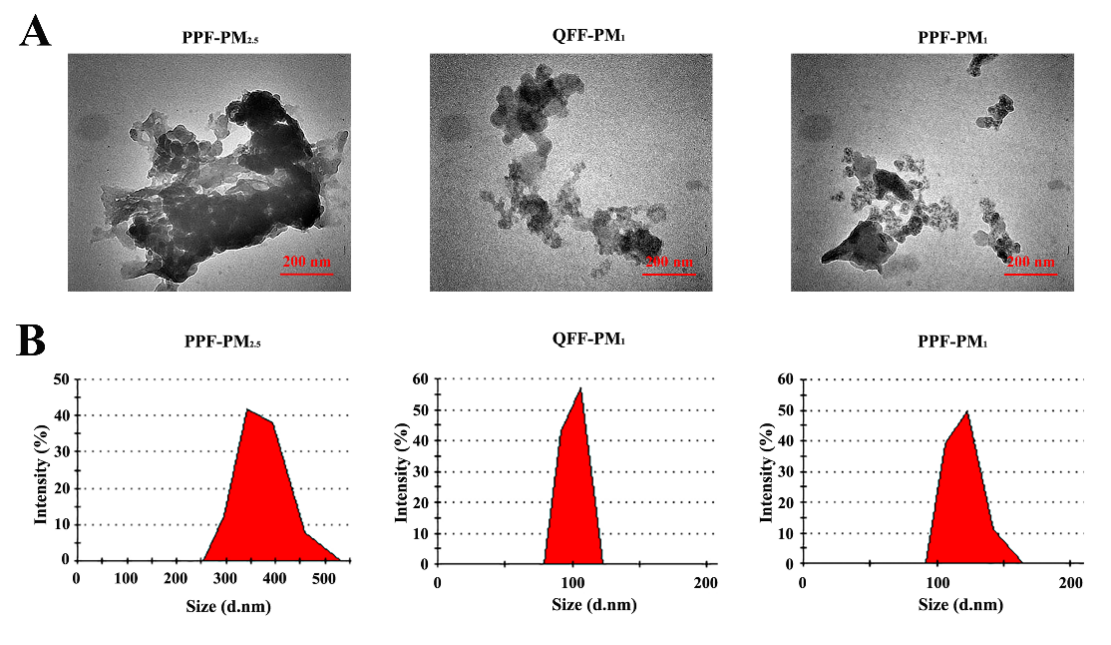


**Fig. S13.** Characterization of airborne fine particles. (A) Representative TEM images of PPF-PM_2.5_, QFF-PM_1_, and PPF-PM_1_. (B) Hydrodynamic diameters of PPF-PM_2.5_, QFF-PM_1_, and PPF-PM_1_ in Milli-Q water.


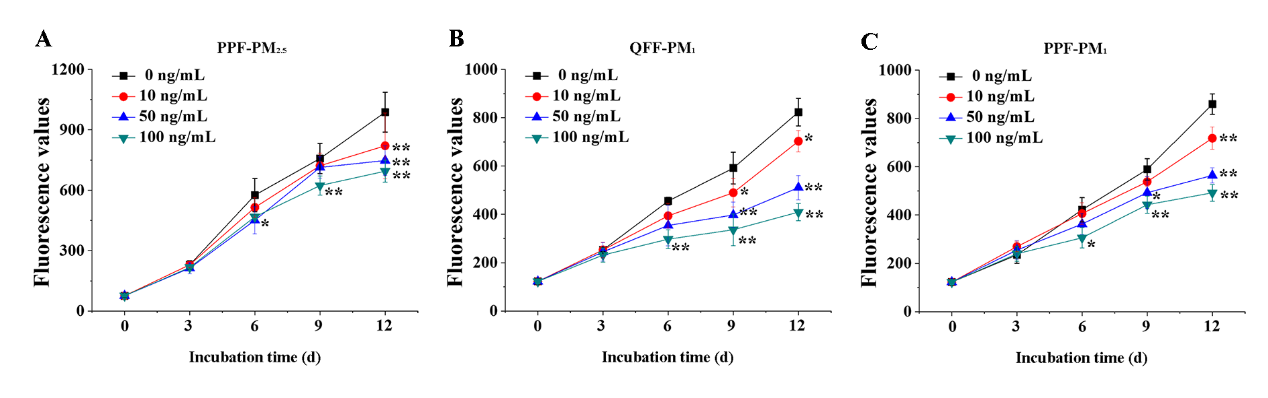


**Fig. S14.** Growth curves of megakaryocytes in different PM exposure groups. (A) PPF-PM_2.5_, (B) QFF-PM_1_ and (C) PPF-PM_1_. **p* < 0.05, or ***p* < 0.01 versus the corresponding controls.


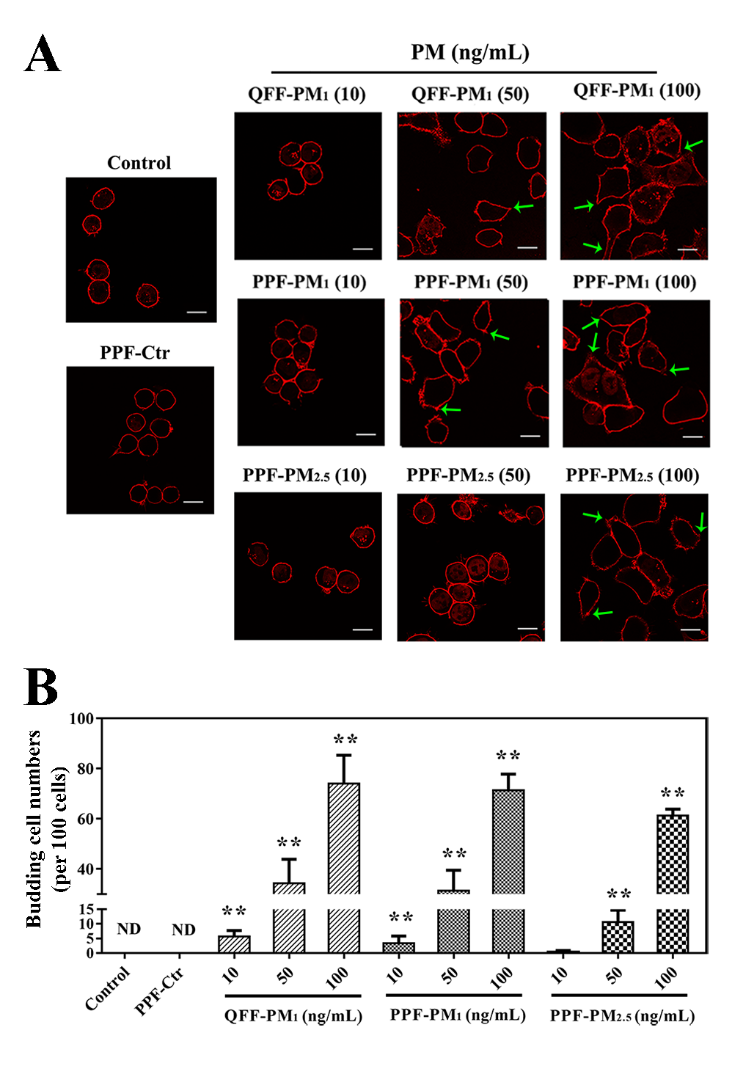


**Fig. S15.** The effects of different PM samples on thrombopoiesis from megakaryocytes. (A) The images for different PM-treated megakaryocytes with wheat germ agglutinin (WGA) staining. The exposure concentrations were 0, 10, 50, and 100 ng/mL for PM samples, and the duration was 12 d. The green arrows denote the budding cells. Scale bar equals to 15 μm. (B) Quantitative analysis of thrombopoiesis by counting the budding cell numbers in different groups (*n* = 4). **p* < 0.05, or ***p* < 0.01 versus the control. PPF-Ctr was evaluated as PM-free PPF filter control.


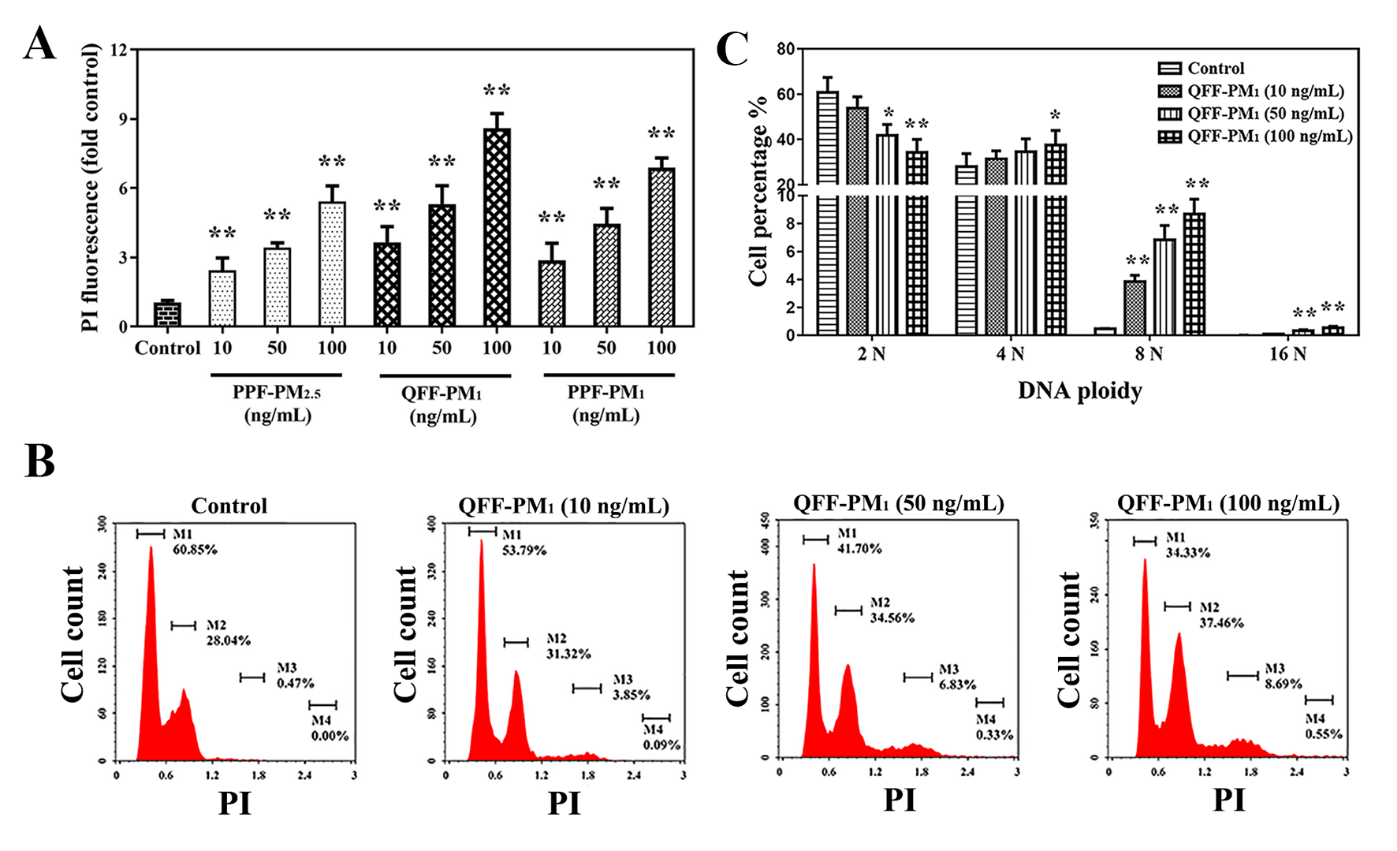


**Fig. S16.** The effects of different PMs on DNA ploidy in megakaryocytes. (A) The DNA contents quantified by PI staining in different groups. ***p* < 0.01 versus the control. (B) Flow cytometry analysis of DNA ploidy change in megakaryocytes treated with different concentrations of QFF-PM_1_. M1, M2, M3, and M4 refer to the 2 N, 4 N, 8 N, and 16 N ploidy, respectively. (C) The quantitative analysis of DNA ploidy changes in megakaryocytes treated with different PM samples (*n* = 4). **p* < 0.05, or ***p* < 0.01 versus the corresponding controls.


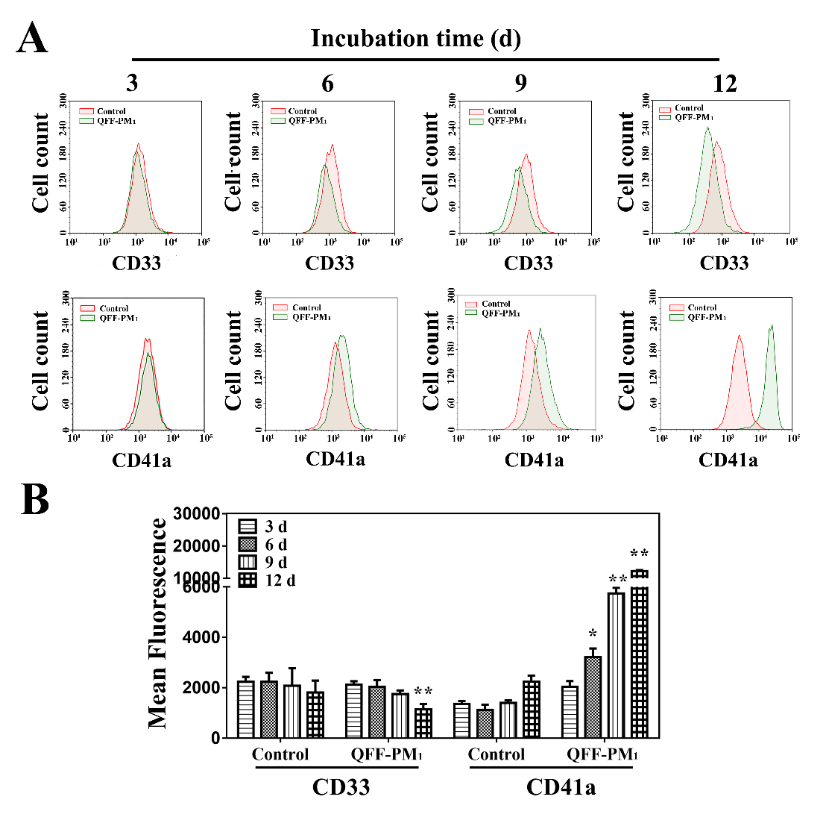


**Fig. S17.** The effect of QFF-PM_1_ treatment on expressions of CD33 and CD41a in megakaryocytes. (A) Time courses for the expressions of CD33 and CD41a in megakaryocytes. The exposure concentration of QFF-PM_1_ was 100 ng/mL. (B) The quantitative analysis of CD33 and CD41a expressions in megakaryocytes. **p* < 0.05, and ***p* < 0.01 versus the corresponding controls.


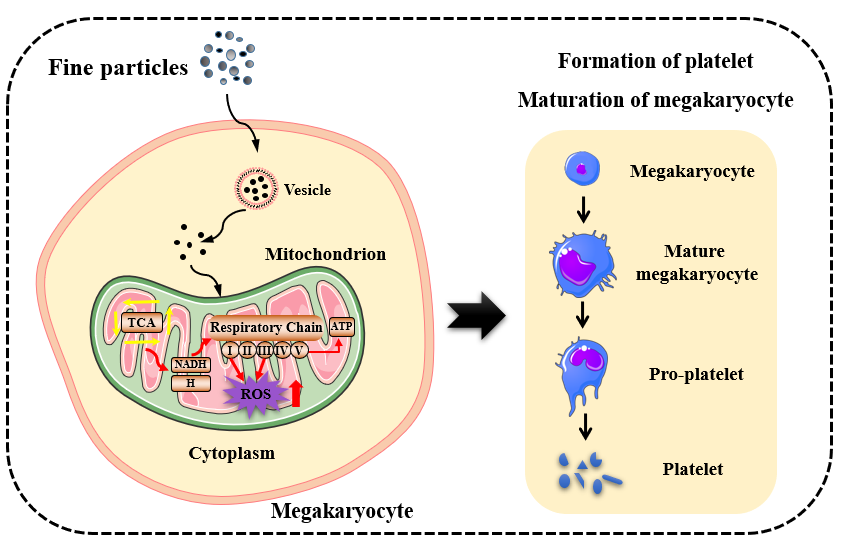


**Fig. S18.** The illustration of thrombopoiesis upon PM exposure and the underlying mechanism.

**Table S1.** The numbers of differentially expressed proteins in megakaryocytes (*p* value < 0.05 and |log_2_ ratio| ≥ 1).

| Protein Number | All test proteins | Interacting proteins | Differentially expressed proteins | Up-regulation | Down-regulation |
| --- | --- | --- | --- | --- | --- |
| PM_2.5_ *vs* Control | 5017 | 3962 | 695 | 337 | 358 |

**Table S2.** The differentially expressed proteins correlated with megakaryocyte differentiation.

| **NO.** | **Protein ID** | **Protein name** | **Gene name** | **MW [kDa]** | **Fold change** | ***p*-**  **value** |
| --- | --- | --- | --- | --- | --- | --- |
| 1 | P27144 | Adenylate kinase 4, mitochondrial | AK4 | 25.268 | 10.579 | 0.008 |
| 2 | Q5VTL8 | Pre-mRNA-splicing factor 38B | PRPF38B | 64.467 | 6.107 | 0.010 |
| 3 | O43678 | NADH dehydrogenase [ubiquinone] | NDUFA2 | 10.921 | 5.578 | 0.021 |
| 4 | P14854 | Cytochrome C oxidase subunit 6B1 | COX6B1 | 10.192 | 4.520 | 0.015 |
| 5 | P48426 | Phosphatidylinositol 5-phosphate 4-kinase type-2 alpha | PIP4K2A | 46.224 | 4.475 | 0.039 |
| 6 | Q9UBC3 | DNA (cytosine-5)-methyltransferase 3B | DNMT3B | 95.750 | 4.407 | 0.013 |
| 7 | Q96J01 | THO complex subunit 3 | THOC3 | 38.771 | 4.269 | 0.000 |
| 8 | X6RAL5 | Histone deacetylase complex subunit | SAP18 | 19.526 | 3.974 | 0.000 |
| 9 | P35658 | Nuclear pore complex protein Nup214 | NUP214 | 213.620 | 3.963 | 0.004 |
| 10 | P47985 | Mitochondrial cytochrome b-c1 complex subunit | UQCRFS1 | 29.668 | 3.917 | 0.001 |
| 11 | O15371 | Eukaryotic translation initiation factor 3 subunit D | EIF3D | 63.972 | 3.818 | 0.026 |
| 12 | O94906 | Pre-mRNA-processing factor 6 | PRPF6 | 106.920 | 3.745 | 0.028 |
| 13 | P30084 | Enoyl-CoA hydratase, mitochondrial | ECHS1 | 31.387 | 3.510 | 0.005 |
| 14 | Q14692 | Ribosome biogenesis protein BMS1 homolog | BMS1 | 145.810 | 3.279 | 0.003 |
| 15 | P51970 | NADH dehydrogenase [ubiquinone] 1 alpha subunit 8 | NDUFA8 | 20.105 | 3.253 | 0.002 |
| 16 | P38159 | RNA-binding motif protein, X chromosome | RBMX | 42.331 | 3.216 | 0.011 |
| 17 | P21912 | Mitochondrial succinate dehydrogenase [ubiquinone] iron-sulfur subunit | SDHB | 31.629 | 3.193 | 0.049 |
| 18 | Q13428 | Treacle protein | TCOF1 | 152.100 | 3.123 | 0.004 |
| 19 | Q9Y277 | Voltage-dependent anion-selective channel protein 3 | VDAC3 | 30.658 | 3.100 | 0.041 |
| 20 | P12270 | Nucleoprotein TPR | TPR | 267.290 | 2.998 | 0.000 |
| 21 | Q96AP7 | Endothelial cell-selective adhesion molecule | ESAM | 41.176 | 2.941 | 0.020 |
| 22 | P20700 | Lamin-B1 | LMNB1 | 66.408 | 2.824 | 0.001 |
| 23 | P16885 | Phosphoinositide phospholipase C | PLCG2 | 147.870 | 2.663 | 0.047 |
| 24 | O60476 | Mannosyl-oligosaccharide 1,2-alpha-mannosidase IB | MAN1A2 | 73.003 | 2.617 | 0.046 |
| 25 | P49792 | E3 SUMO-protein ligase RanBP2 | RANBP2 | 358.200 | 2.551 | 0.004 |
| 26 | P05556 | Integrin beta-1 | ITGB1 | 88.414 | 2.523 | 0.037 |
| 27 | Q9BW27 | Nuclear pore complex protein Nup85 | NUP85 | 75.019 | 2.496 | 0.008 |
| 28 | G8JLG1 | Structural maintenance of chromosomes protein | SMC1A | 140.86 | 2.495 | 0.009 |
| 29 | G3V3A4 | SNW domain-containing protein 1 | SNW1 | 65.391 | 2.466 | 0.002 |
| 30 | P54886 | Delta-1-pyrroline-5-carboxylate synthase | ALDH18A1 | 87.301 | 2.371 | 0.005 |
| 31 | Q9P013 | Spliceosome-associated protein CWC15 homolog | CWC15 | 26.624 | 2.369 | 0.005 |
| 32 | P52272 | Heterogeneous nuclear ribonucleoprotein M | HNRNPM | 77.515 | 2.264 | 0.014 |
| 33 | O00116 | Alkyldihydroxyacetonephosphate synthase, peroxisomal | AGPS | 72.911 | 2.256 | 0.049 |
| 34 | P22695 | Mitochondrial cytochrome b-c1 complex subunit 2 | UQCRC2 | 48.442 | 2.246 | 0.032 |
| 35 | Q92769 | Histone deacetylase 2 | HDAC2 | 55.364 | 2.187 | 0.027 |
| 36 | O15042 | U2 snRNP-associated SURP motif-containing protein | U2SURP | 118.290 | 2.187 | 0.007 |
| 37 | P23219 | Prostaglandin G/H synthase 1 | PTGS1 | 68.686 | 2.185 | 0.024 |
| 38 | O95831 | Apoptosis-inducing factor 1, mitochondrial | AIFM1 | 66.900 | 2.177 | 0.000 |
| 39 | Q6P2Q9 | Pre-mRNA-processing-splicing factor 8 | PRPF8 | 273.600 | 2.168 | 0.009 |
| 40 | Q03252 | Lamin-B2 | LMNB2 | 69.948 | 2.074 | 0.003 |
| 41 | Q9NPE3 | H/ACA ribonucleoprotein complex subunit 3 | NOP10 | 7.706 | 2.055 | 0.016 |
| 42 | Q06187 | Tyrosine-protein kinase BTK | BTK | 76.280 | 0.488 | 0.015 |
| 43 | Q00610 | Clathrin heavy chain | CLTC | 192.060 | 0.472 | 0.025 |
| 44 | Q6XQN6 | Nicotinate phosphoribosyltransferase | NAPRT | 57.578 | 0.461 | 0.015 |
| 45 | P06396 | Gelsolin | GSN | 82.525 | 0.450 | 0.015 |
| 46 | A6NMQ1 | DNA polymerase;DNA polymerase alpha catalytic subunit | POLA1 | 166.460 | 0.442 | 0.029 |
| 47 | P33316 | Deoxyuridine 5-triphosphate nucleotidohydrolase, mitochondrial | DUT | 26.563 | 0.434 | 0.028 |
| 48 | P23396 | 40S ribosomal protein S3 | RPS3 | 26.688 | 0.413 | 0.012 |
| 49 | P62280 | 40S ribosomal protein S11 | RPS11 | 18.431 | 0.405 | 0.017 |
| 50 | P62424 | 60S ribosomal protein L7a | RPL7A | 29.995 | 0.391 | 0.000 |
| 51 | P19623 | Spermidine synthase | SRM | 33.824 | 0.376 | 0.031 |
| 52 | P18077 | 60S ribosomal protein L35a | RPL35A | 12.538 | 0.373 | 0.031 |
| 53 | Q9Y6A5 | Transforming acidic coiled-coil-containing protein 3 | TACC3 | 90.359 | 0.368 | 0.022 |
| 54 | O14980 | Exportin-1 | XPO1 | 123.380 | 0.350 | 0.003 |
| 55 | P62249 | 40S ribosomal protein S16 | RPS16 | 16.445 | 0.339 | 0.006 |
| 56 | P08575 | Receptor-type tyrosine-protein phosphatase C | PTPRC | 147.480 | 0.326 | 0.005 |
| 57 | P62269 | 40S ribosomal protein S18 | RPS18 | 17.718 | 0.322 | 0.005 |
| 58 | P04899 | Guanine nucleotide-binding protein G(i) subunit alpha-2 | GNAI2 | 40.450 | 0.308 | 0.011 |
| 59 | P18124 | 60S ribosomal protein L7 | RPL7 | 29.225 | 0.292 | 0.043 |
| 60 | Q96G03 | Phosphoglucomutase-2 | PGM2 | 68.283 | 0.292 | 0.011 |
| 61 | P25398 | 40S ribosomal protein S12 | RPS12 | 14.515 | 0.286 | 0.013 |
| 62 | F8VPD4 | CAD protein | CAD | 236.02 | 0.274 | 0.001 |
| 63 | P23526 | Adenosylhomocysteinase | AHCY | 47.716 | 0.273 | 0.011 |
| 64 | P62750 | 60S ribosomal protein L23a | RPL23A | 17.695 | 0.271 | 0.002 |
| 65 | P63261 | Actin, cytoplasmic 2 | ACTG1 | 41.792 | 0.267 | 0.002 |
| 66 | P42224 | Signal transducer and activator of transcription 1-alpha/beta | STAT1 | 87.334 | 0.265 | 0.000 |
| 67 | Q13131 | 5-AMP-activated protein kinase catalytic subunit alpha-1 | PRKAA1 | 64.009 | 0.265 | 0.005 |
| 68 | P07737 | Profilin-1 | PFN1 | 15.054 | 0.235 | 0.019 |
| 69 | P43034 | Platelet-activating factor acetylhydrolase IB subunit alpha | PAFAH1B1 | 46.637 | 0.217 | 0.001 |
| 70 | Q01813 | ATP-dependent 6-phosphofructokinase, platelet type | PFKP | 85.595 | 0.202 | 0.000 |
| 71 | P27348 | 14-3-3 protein theta | YWHAQ | 27.764 | 0.200 | 0.000 |
| 72 | P41091 | Eukaryotic translation initiation factor 2 subunit 3 | EIF2S3 | 51.109 | 0.194 | 0.002 |
| 73 | P62851 | 40S ribosomal protein S25 | RPS25 | 13.742 | 0.181 | 0.001 |
| 74 | P55160 | Nck-associated protein 1-like | NCKAP1L | 128.15 | 0.178 | 0.036 |
| 75 | Q14232 | Translation initiation factor eIF-2B subunit alpha | EIF2B1 | 33.712 | 0.178 | 0.001 |
| 76 | Q06124 | Tyrosine-protein phosphatase non-receptor type 11 | PTPN11 | 68.436 | 0.157 | 0.043 |
| 77 | P62917 | 60S ribosomal protein L8 | RPL8 | 28.024 | 0.143 | 0.006 |
| 78 | P56385 | ATP synthase subunit e, mitochondrial | ATP5I | 7.933 | 0.141 | 0.001 |
| 79 | P16152 | Carbonyl reductase [NADPH] 1 | CBR1 | 30.375 | 0.129 | 0.014 |
| 80 | P52209 | 6-phosphogluconate dehydrogenase, decarboxylating | PGD | 53.139 | 0.100 | 0.000 |
| 81 | P62277 | 40S ribosomal protein S13 | RPS13 | 17.222 | 0.098 | 0.006 |
| 82 | Q86UX7 | Fermitin family homolog 3 | FERMT3 | 75.952 | 0.095 | 0.000 |
| 83 | P62906 | 60S ribosomal protein L10a | RPL10A | 24.831 | 0.089 | 0.003 |
| 84 | Q9Y3F4 | Serine-threonine kinase receptor-associated protein | STRAP | 38.438 | 0.045 | 0.032 |

**Table S3.** Statistical analysis of the influencing factors for PM-induced thrombopoiesis.

| Factor | DF | SS | MS | F Value | *p* value |
| --- | --- | --- | --- | --- | --- |
| Exposure concentration (EC) | 2 | 27666.889 | 13833.444 | 297.138 | 0.000001 |
| Filter type (FT) | 1 | 61.361 | 61.361 | 1.318 | 0.262262 |
| PM size | 1 | 1144.694 | 1144.694 | 24.588 | 0.000046 |
| EC*PM size | 2 | 633.556 | 316.778 | 6.804 | 0.004561 |
| EC*FS*PM size | 5 | 53.139 | 10.628 | 0.228 | 0.946505 |

Note: Three-way ANOVA method was used. DF, degree of freedom. SS, the variation from different variables. MS, the mean square from different variables.

**Table S4.** The influence of PM sampling filter type on thrombopoiesis.

| PM | Exposure concentration  (ng/mL) | Filter type | | *p* value |
| --- | --- | --- | --- | --- |
|  |  | QFF | PPF |  |
| PM_1_ | 10 | 5.67 ± 1.70 | 3.33 ± 2.05 | 0.9999 |
| PM_1_ | 50 | 34.33 ± 7.76 | 31.33 ± 6.65 | 0.9999 |
| PM_1_ | 100 | 74.00 ± 9.27 | 71.33 ± 5.31 | 0.9999 |
| PM_2.5_ | 10 | 2.33 ± 1.25 | 0.33 ± 0.47 | 0.9999 |
| PM_2.5_ | 50 | 9.33 ± 3.68 | 10.67 ± 3.30 | 0.9999 |
| PM_2.5_ | 100 | 68.33 ± 10.78 | 61.33 ± 2.05 | 0.9773 |

Note: The Tukey method was used to analyze the effect of sampling filter type on thrombopoiesis given the same PM size and exposure dose.

**Table S5.** The influence of PM size on thrombopoiesis.

| Filter type | Exposure dose (ng/mL) | PM | | | *p* value |
| --- | --- | --- | --- | --- | --- |
|  |  | PM_1_ | PM_2.5_ | |  |
| QFF | 10 | 5.67 ± 2.08 | | 2.33 ± 1.53 | 1.0000 |
| QFF | 50 | 34.33 ± 9.50 | | 9.33 ± 4.51** | 0.0067 |
| QFF | 100 | 74.00 ± 11.36 | | 68.33 ± 13.20 | 0.9956 |
| PPF | 10 | 3.33 ± 2.52 | | 0.33 ± 0.58 | 1.0000 |
| PPF | 50 | 31.33 ± 8.14 | | 10.67 ± 4.04* | 0.0399 |
| PPF | 100 | 71.33 ± 6.51 | | 61.33 ± 2.52 | 0.8056 |

Note: The Tukey method was used to analyze the effect of PM size on thrombopoiesis given the same PM exposure dose and sampling filter. **p* < 0.05, or ***p* < 0.01.

**Table S6.** Statistical analysis for the effect of PM size on PM-promoted megakaryocytic maturation.

| Index | PM | | *p* value |
| --- | --- | --- | --- |
|  | QFF-PM_1_ | QFF-PM_2.5_ |  |
| Total DNA | 8.59 ± 0.52 | 6.38 ± 0.29** | 0.0063 |
| 2N | 34.33 ± 5.68 | 37.11 ± 5.24 | 0.5670 |
| 4N | 37.46 ± 6.54 | 34.18 ± 4.61 | 0.5169 |
| 8N | 8.69 ± 1.06 | 7.65 ± 1.23 | 0.3295 |
| 16N | 0.55 ± 0.096 | 0.42 ± 0.075 | 0.1383 |
| CD33 | 1144 ± 213.45 | 1096 ± 143.11 | 0.7625 |
| CD41a | 12254 ± 312.54 | 11287 ± 469.87* | 0.0412 |

Note: One-way ANOVA method was used to statistically analyze the differences of total DNA (indicated by PI fluorescence), cell percentages of 2N, 4N, 8N, 6N and the expression of CD33 and CD41a (indicated by the corresponding fluorescence intensities) between PM_1_ and PM_2.5_ exposure groups. ***p* < 0.01 or **p* < 0.05.

**Table S7.** Pair primer sequences for quantitative polymerase chain reaction (*q*PCR) analysis.

| **Gene** | **Primer** | **Sequence (5’→3’)** |
| --- | --- | --- |
| **NDUFA2** | Forward | AGGGCGTCAGGGACTTCATT |
|  | Reverse | CTCTTGGCCAAATGCGTAGC |
| **NDUFA8** | Forward | GCCGCTGGGAAGAGAAAGAT |
|  | Reverse | CTGCTGTTTGCGACAGTGAC |
| **SDHB** | Forward | ATGTGGCCCCATGGTATTGG |
|  | Reverse | TGGTGTCAATCCTTCGGGTG |
| **UQCRFS1** | Forward | CCGCCGCCTTGAAGTTTTAG |
|  | Reverse | AACTGGGTGACGGCATTCTT |
| **UQCRC2** | Forward | TCAATGTCACCACAGCACCA |
|  | Reverse | ATTAGCCAAGGCATTCCGGT |
| **COX6B1** | Forward | TACAAGACCGCCCCTTTTGA |
|  | Reverse | TATCGCCTCCTTTAGCGGTC |
| **GAPDH** | Forward | ATCTTCCAGGAGCGAGATCC |
|  | Reverse | CTGCAAATGAGCCCCAGCCT |

Reference

1. Jin X, Ma Q, Sun Z, Yang X, Zhou Q, Qu G, et al. Airborne fine particles induce hematological effects through regulating the crosstalk of the kallikrein-kinin, complement, and coagulation systems. Environ Sci Technol. 2019;53 5:2840-51. <http://dx.doi.org/10.1021/acs.est.8b05817> <https://www.ncbi.nlm.nih.gov/pubmed/30742439>.
